# Responses of Soil Carbon and Nitrogen Cycling Gene Abundances to Winter Green Manure Incorporation in a Ratoon Rice System

**DOI:** 10.3390/plants15172578

**Published:** 2026-08-24

**Authors:** Pufan Shao, Sheng Chen, Shumin Jia, Mianchun Shi, Qiwen Hou, Zhangzhen Yang, Liusheng Zhong, Zhixiong Yuan, Cheng Huang, Zhiqiang Fu, Pan Long, Ying Xu

**Affiliations:** 1 College of Agronomy, Hunan Agricultural University, Changsha 410128, China; 2 Yuelushan Laboratory, Changsha 410128, China

**Keywords:** ratoon rice, green manure, rapeseed, Chinese milk vetch, nitrogen cycling, functional genes, ammonia oxidizers, denitrifiers

## Abstract

Winter green manure incorporation is widely practised in southern China, yet its effect on soil carbon and nitrogen cycling gene abundances across a full ratoon rice cycle remains unclear. A field experiment combined three cropping systems differing in winter management (winter fallow–ratoon rice, FA; rapeseed–ratoon rice, RA; and Chinese milk vetch–ratoon rice, MV) with two ratoon rice varieties (Yliangyou 911 and Liangyou 6326). Soil properties and functional gene abundances were measured at three soil depths and three sampling stages, and theoretical grain yield was determined in the main and ratoon seasons. Green manure incorporation did not change the bulk physicochemical properties of the soil as a general effect but altered its labile carbon and nitrogen pools. Dissolved organic carbon under RA and MV was, respectively, 24.28% and 16.15% higher than under FA, and ammonium nitrogen under MV in the ratoon season was up to 193.04% higher than under FA, exceeding RA throughout the profile. The functional genes responded in contrasting directions. *pmoA*, *mcrA*, *nirK* and *nosZ* were lower under green manure (*pmoA* 20.00% and 14.53% lower under RA and MV), whereas AOA *amoA*, *cnorB* and *nirS* were higher (AOA *amoA* 0.42 and 0.55 log units higher), with the clearest shifts occurring in the ratoon season and the surface soil. Correlation, redundancy and path analyses indicated that the gene–environment associations were linked mainly to ammonium nitrogen, dissolved organic carbon and nitrate nitrogen, and that winter treatment was associated with yield indirectly, most closely through the soil carbon pool (standardized total effect 0.34–0.57), rather than acting on yield directly.

## 1. Introduction

Ratoon rice, in which a second grain harvest is taken from tillers that regenerate on the stubble of the main crop, has become an increasingly important cropping system in the middle and lower Yangtze region [1]. A single transplanting yields two harvests, and the ratoon season is grown with roughly 30% less labour and with marked savings in seed, water and fertiliser relative to a second transplanted crop [2,3]. The arrangement suits areas whose thermal resources exceed the requirement of single-season rice but fall short of those needed for double cropping, a niche estimated to cover several million hectares of Chinese paddy land [3,4]. Yield in the ratoon season is set largely during the main season: the regenerating buds draw on assimilates and nutrients left in the stubble, and light and nutrient conditions during main-crop grain filling strongly govern the ratooning ability of the stubble [5]. Because the two harvests share one continuous nutrient supply and one microbial community, the state in which the soil enters and leaves the main season is central to the productivity of the whole system.

The winter fallow that precedes the main season is the point at which that soil state can be managed. Across much of southern China paddies lie bare from mid-October to late April, and returning the biomass of a winter cover crop to the soil before transplanting is a long-standing way of using this idle period [6,7]. Chinese milk vetch (*Astragalus sinicus* L.), a leguminous green manure with a low carbon-to-nitrogen ratio, decomposes rapidly once incorporated, releasing most of its nitrogen within the first four months of the rice season [8,9]. Long-term experiments in the double-rice belt of Jiangxi have shown that milk vetch incorporation, combined with a modest reduction in mineral fertiliser, raises early- and late-rice yields by several percent while improving soil organic matter and nutrient-use efficiency [10]. Rapeseed (*Brassica napus* L.), by contrast, is a non-leguminous winter crop whose residues carry a higher carbon-to-nitrogen ratio and decompose more slowly; the rice–rapeseed rotation is itself one of the major cropping systems of the region [11,12], and in double-rice soils, rapeseed incorporation has been reported to raise gross nitrogen immobilisation and lower gross mineralisation, so that nitrogen is held in the microbial biomass for longer before net release [13]. The amount and timing of nitrogen made available to the crop therefore differ between the two residue types, and their consequences for the soil nitrogen and carbon cycles need not coincide.

The soil processes that link residue return to grain yield are driven by functional microbial groups, and the size of each group can be tracked through a marker gene. In flooded paddies, nitrogen is transformed through nitrification, mediated by the ammonia monooxygenase gene (*amoA*) of ammonia-oxidising archaea (AOA) and bacteria (AOB), and through the sequential steps of denitrification, mediated by the nitrate reductase gene (*narG*), the nitrite reductase genes (*nirK* and *nirS*), the nitric oxide reductase gene (*cnorB*) and the nitrous oxide reductase gene (*nosZ*) [14,15]. Carbon turnover under the alternating oxic and anoxic conditions of paddy soil is reflected in the abundances of methanogens carrying the methyl-coenzyme M reductase gene (*mcrA*) and methanotrophs carrying the particulate methane monooxygenase gene (*pmoA*) [16]. Residue incorporation shifts the supply of labile carbon, ammonium and nitrate on which these groups depend, and studies in single- and double-cropped rice have reported that straw or green manure return alters denitrifier and methanogen gene abundances, though the direction of the change varies with soil and residue type [17]. Working across several paddy sites in south China, Li et al. [18] found that green manuring changed the abundances of nitrogen-cycling genes, that *nirK* and *nosZ* were negatively correlated with improving soil properties, and that *nirK* was the single most influential gene for rice yield among those examined. Such findings suggest that residue return may act on yield through a chain that runs from soil properties to functional genes and on to nutrient availability, rather than through the amendment itself, but how this chain behaves when the disturbance is a winter cover crop, and whether it holds through both the main and ratoon seasons and down the soil profile, has rarely been resolved within one experiment.

Most of what is known about cover-crop residue return in rice comes from single- or double-season systems [19], and the results transfer poorly to ratoon rice, a system in which one fertilisation and one stubble must carry two harvests [20,21]. Few studies have followed soil physicochemical properties, carbon- and nitrogen-cycling gene abundances and grain yield together across a complete ratoon cycle, and fewer still have set a leguminous against a non-leguminous winter residue in this setting, despite the difference in their decomposition dynamics. Whether functional gene abundances near the surface, where methanotrophs and denitrifiers are usually most numerous [22], respond differently from those deeper in the profile also remains unclear.

Therefore, a field experiment spanning the winter incorporation period, the main season and the ratoon season was conducted, combining three winter treatments (fallow, rapeseed incorporation and milk vetch incorporation) with two ratoon rice cultivars, YLY911 and LY6326. Soil physicochemical properties and the abundances of carbon- and nitrogen-cycling genes were measured at three depths and three sampling stages, and their interrelationships were examined by Spearman’s rank correlation, redundancy analysis and partial least squares path modelling. We hypothesised that (i) incorporation of rapeseed or milk vetch would raise theoretical grain yield in both the main and ratoon seasons relative to winter fallow; (ii) the two residues, differing in carbon-to-nitrogen ratio, would alter the abundances of specific nitrogen- and carbon-cycling genes in distinguishable ways; and (iii) the effect of winter treatment on theoretical grain yield would be indirect, transmitted through changes in soil physicochemical properties, functional gene abundances and soil carbon and nitrogen pools, rather than acting on yield directly.

## 2. Results

### 2.1. Theoretical Grain Yield

**Figure 1:**
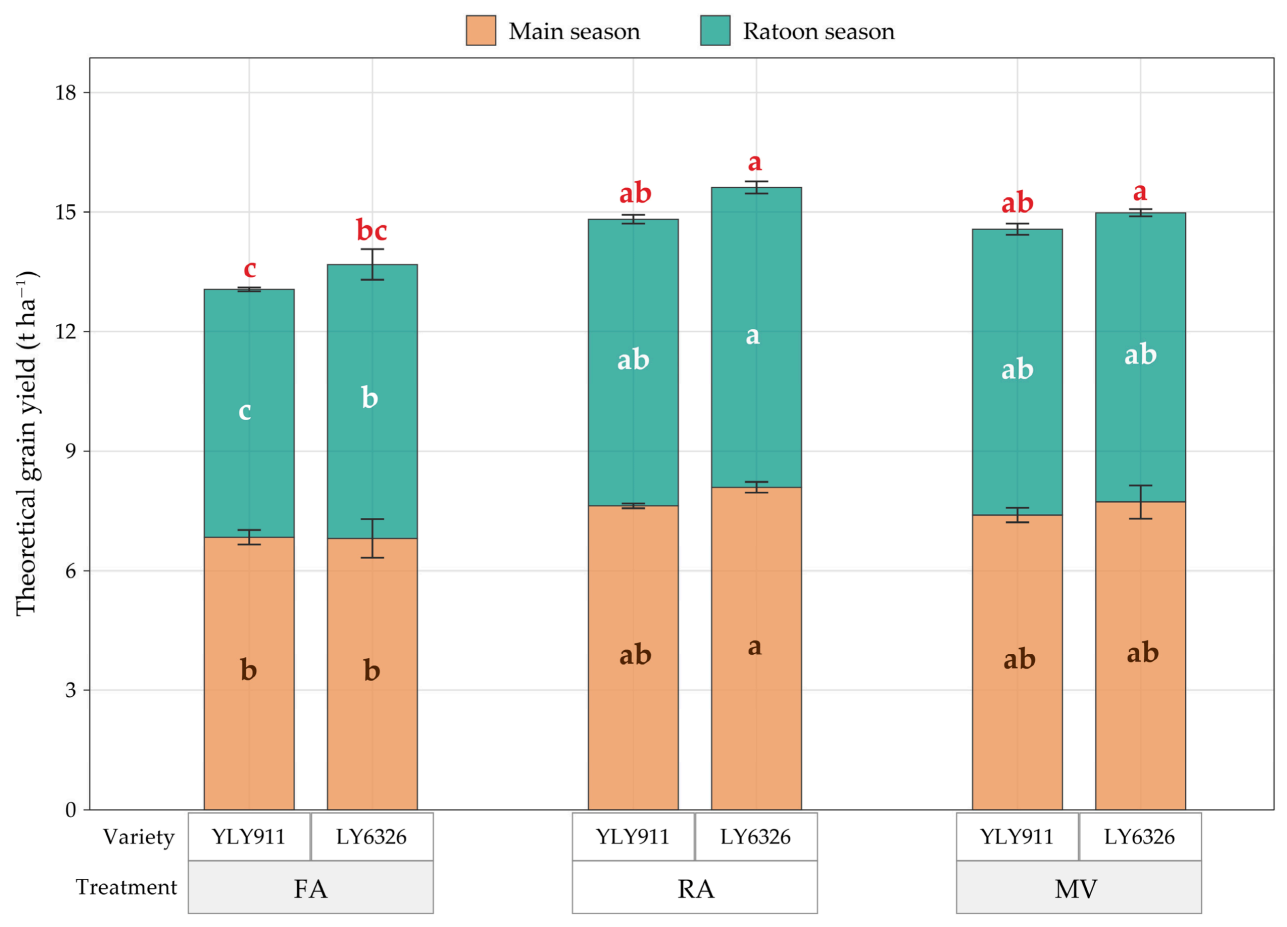
Theoretical grain yield of two ratoon rice varieties in the main and ratoon seasons under three cropping systems. FA, winter fallow–ratoon rice; RA, rapeseed–ratoon rice; MV, Chinese milk vetch–ratoon rice; YLY911, Yliangyou 911; LY6326, Liangyou 6326. The total bar height represents the annual yield (main season + ratoon season). Data are presented as mean ± standard error (SE). Different lowercase letters within the main-season segments, within the ratoon-season segments, and above the bars indicate significant differences among treatment combinations for the main season, the ratoon season, and the annual yield, respectively, at *p* < 0.05 according to the least significant difference (LSD) test.

Theoretical grain yields of the two ratoon rice varieties in the main and ratoon seasons under the three cropping systems are presented in Figure 1. Season, green manure and rice variety all had significant main effects on theoretical grain yield, whereas none of their interactions was significant (Table S1). In the main season, theoretical grain yields under winter fallow–ratoon rice (FA) were 6.84 and 6.81 t ha^−1^ for Yliangyou 911 (YLY911) and Liangyou 6326 (LY6326), the lowest among all combinations (Figure 1). The corresponding means under rapeseed–ratoon rice (RA) and Chinese milk vetch–ratoon rice (MV) were 7.85 and 7.55 t ha^−1^, 15.04% and 10.66% higher than under FA, and RA–LY6326 reached 8.09 t ha^−1^, significantly higher than both combinations under FA (Figure 1). Treatment differentiation was more pronounced in the ratoon season, in which FA–YLY911 gave the lowest value of 6.21 t ha^−1^, 21.20% lower than the highest combination RA–LY6326 (7.52 t ha^−1^), while the means under RA and MV were 12.60% and 10.40% higher than under FA (Figure 1). For the annual yield, FA–YLY911 was the lowest at 13.05 t ha^−1^ and RA–LY6326 the highest at 15.61 t ha^−1^, a difference of 2.56 t ha^−1^; annual yields under RA and MV exceeded those under FA by 13.85% and 10.53% (Figure 1). Averaged across cropping systems and seasons, LY6326 produced an annual yield of 14.76 t ha^−1^, 4.31% higher than YLY911 (Figure 1).

### 2.2. Soil Physicochemical Properties

**Figure 2:**
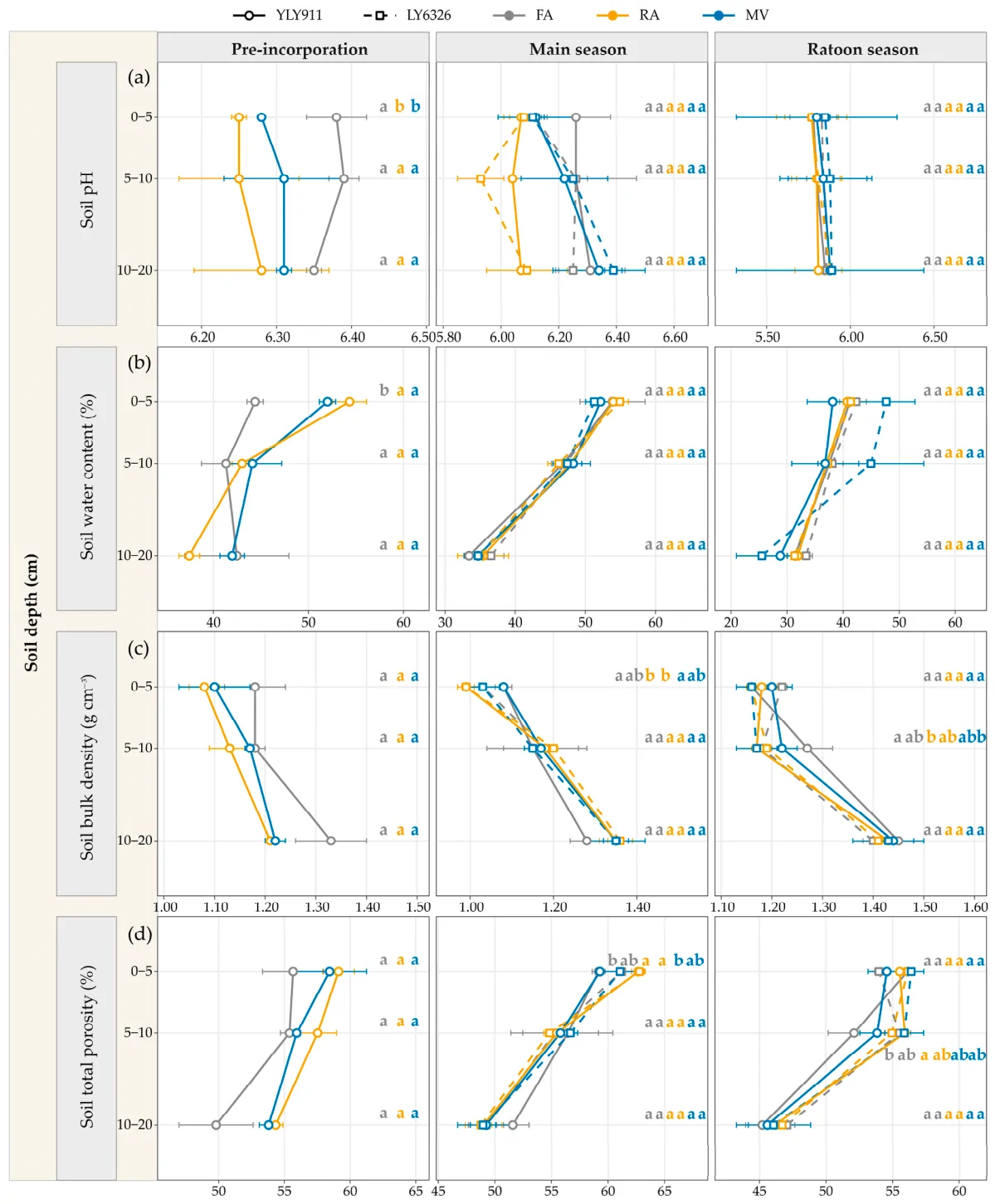
Vertical distribution of (**a**) soil pH, (**b**) soil water content, (**c**) soil bulk density and (**d**) soil total porosity under three cropping systems at three sampling stages. Treatment and variety abbreviations are defined in Figure 1. Soil samples were collected before residue incorporation (pre-incorporation) and at maturity of the main season and the ratoon season. Data are presented as mean ± SE. Within each panel and soil depth, different lowercase letters indicate significant differences among treatments at *p* < 0.05 according to the LSD test. Letters are arranged from left to right in the order FA, RA and MV, with YLY911 preceding LY6326 within each cropping system.

The vertical distributions of soil pH, soil water content (SWC), soil bulk density (BD) and soil total porosity (STP) under the three cropping systems are shown in Figure 2. Season had highly significant effects on all four properties, and soil depth on SWC, BD and STP, with significant season × depth interactions for these three properties, whereas neither green manure nor rice variety, nor any other interaction, was significant (Table S2). Before residue incorporation, soil pH in the 0–5 cm layer was 6.38 under FA, significantly higher than under RA (6.25) and MV (6.28), while SWC under FA was 44.39%, significantly lower than under RA (54.33%) and MV (52.02%), which exceeded FA by 22.38% and 17.17%, respectively; no significant differences were detected in the 5–10 and 10–20 cm layers (Figure 2a,b). In the main and ratoon seasons, neither soil pH nor SWC differed significantly among treatments at any depth (Figure 2a,b). Soil pH declined from 6.17 in the main season to 5.83 in the ratoon season, with comparable decreases at all depths, whereas the seasonal decline in SWC diminished with depth: values fell from 53.38%, 47.29% and 34.97% to 41.90%, 38.61% and 30.39% at 0–5, 5–10 and 10–20 cm, corresponding to decreases of 21.50%, 18.36% and 13.11% (Figure 2a,b). BD increased and STP decreased with soil depth; in the main season, BD rose from 1.03 g cm^−3^ at 0–5 cm to 1.34 g cm^−3^ at 10–20 cm, with STP falling from 61.04% to 49.47% (Figure 2c,d). In the 0–5 cm layer of the main season, BD under both combinations of RA was 0.99 g cm^−3^, 8.46% lower than the 1.08 g cm^−3^ recorded for FA–YLY911 and MV–YLY911, and their STP values of 62.69% and 62.78% were significantly higher than the 59.18% and 59.29% of these two combinations (Figure 2c,d). In the 5–10 cm layer of the ratoon season, FA–YLY911 had the highest BD (1.27 g cm^−3^), significantly exceeding RA–YLY911 and MV–LY6326 (both 1.17 g cm^−3^) by 7.90%; no significant differences among combinations were detected at the remaining depths (Figure 2c,d).

### 2.3. Soil Carbon and Nitrogen Pools

**Figure 3:**
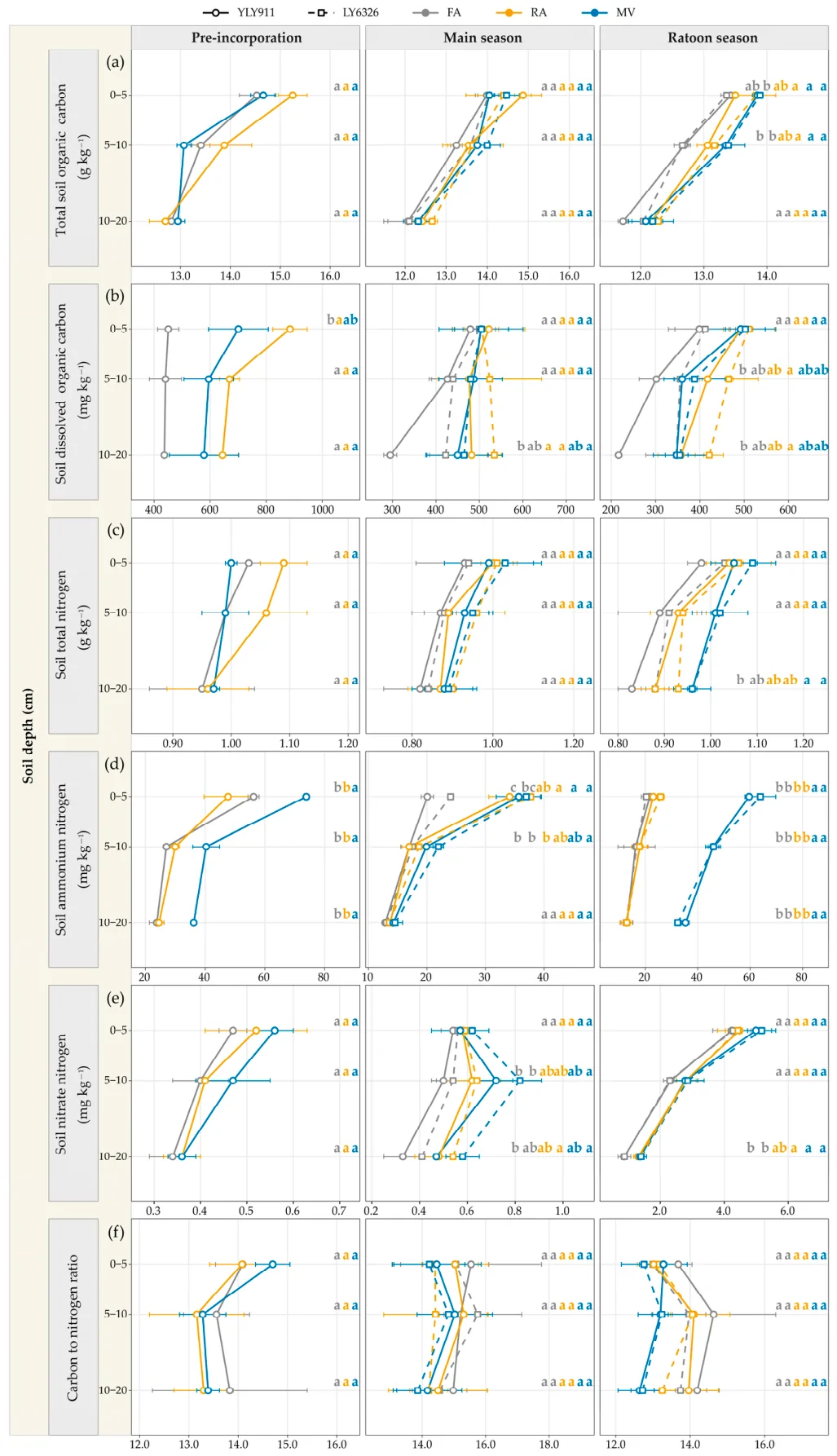
Vertical distribution of (**a**) total soil organic carbon, (**b**) soil dissolved organic carbon, (**c**) soil total nitrogen, (**d**) soil ammonium nitrogen, (**e**) soil nitrate nitrogen and (**f**) the carbon to nitrogen ratio under three cropping systems at three sampling stages. Treatment and variety abbreviations are defined in Figure 1. Soil samples were collected before residue incorporation (pre-incorporation) and at maturity of the main season and the ratoon season. Data are presented as mean ± SE. Within each panel and soil depth, different lowercase letters indicate significant differences among treatments at *p* < 0.05 according to the LSD test. Letters are arranged from left to right in the order FA, RA and MV, with YLY911 preceding LY6326 within each cropping system.

The vertical distributions of total soil organic carbon (SOC), soil dissolved organic carbon (DOC), soil total nitrogen (STN), soil ammonium nitrogen (NH_4_^+^–N), soil nitrate nitrogen (NO_3_^−^–N) and the carbon to nitrogen ratio (C/N) under the three cropping systems are shown in Figure 3. In contrast to the physicochemical properties, green manure had highly significant effects on SOC, DOC, STN, NH_4_^+^–N and NO_3_^−^–N but not on C/N, whereas rice variety affected none of the six variables (Table S3).

For the carbon pools, SOC decreased from 13.97 g kg^−1^ at 0–5 cm to 12.20 g kg^−1^ at 10–20 cm, a decline of 12.71%, and mean SOC under RA and MV was 13.30 and 13.31 g kg^−1^, 2.97% and 3.06% higher than the 12.91 g kg^−1^ under FA (Figure 3a). No significant differences among treatments were detected before residue incorporation or in the main season, whereas differentiation appeared in the 0–5 and 5–10 cm layers of the ratoon season, in which SOC under MV reached 13.37 g kg^−1^ at 5–10 cm, 5.50% higher than the 12.68 g kg^−1^ under FA (Figure 3a). Treatment differences were more pronounced for DOC, with means of 475.82 and 444.70 mg kg^−1^ under RA and MV, 24.28% and 16.15% higher than the 382.86 mg kg^−1^ under FA (Figure 3b). Before residue incorporation, DOC in the 0–5 cm layer was 884.87 mg kg^−1^ under RA, 96.29% higher than the 450.80 mg kg^−1^ under FA; in the 10–20 cm layer, FA–YLY911 gave the lowest values in both the main and ratoon seasons (294.81 and 216.68 mg kg^−1^), significantly lower than RA–LY6326 (534.64 and 421.17 mg kg^−1^) (Figure 3b).

For the nitrogen pools, mean STN was highest under MV (0.98 g kg^−1^), 8.96% higher than under FA (0.90 g kg^−1^), with significant differences confined to the 10–20 cm layer of the ratoon season, where both combinations under MV (0.96 g kg^−1^) significantly exceeded FA–YLY911 (0.83 g kg^−1^) (Figure 3c). NH_4_^+^–N showed the strongest response to green manure, and this response varied with both season and soil depth (Table S3). Before residue incorporation, NH_4_^+^–N under MV was 73.87, 40.33 and 36.15 mg kg^−1^ at 0–5, 5–10 and 10–20 cm, significantly higher than under FA and RA at all three depths and exceeding FA by 31.43% to 51.95% (Figure 3d). In the main season, the differences were largest in the 0–5 cm layer, where RA and MV exceeded FA by 62.89% and 64.77%; they narrowed to 3.17% and 21.25% at 5–10 cm and were no longer significant at 10–20 cm (Figure 3d). Differentiation was most pronounced in the ratoon season, in which NH_4_^+^–N under MV reached 61.78, 45.97 and 33.86 mg kg^−1^ at the three depths, exceeding FA by 193.04%, 178.70% and 163.04%, whereas RA did not differ significantly from FA at any depth (Figure 3d). NO_3_^−^–N was dominated by season and was 5.01 times higher in the ratoon season (2.82 mg kg^−1^) than in the main season (0.56 mg kg^−1^), with the seasonal increase diminishing with depth (7.97, 4.14 and 2.57 times at 0–5, 5–10 and 10–20 cm) (Figure 3e). NO_3_^−^–N under RA and MV exceeded that under FA in the 5–10 and 10–20 cm layers of the main season and in the 10–20 cm layer of the ratoon season, where MV reached 1.39 mg kg^−1^, 58.09% higher than the 0.88 mg kg^−1^ under FA (Figure 3e). C/N was affected only by season, declining from 14.75 in the main season to 13.48 in the ratoon season, with no significant differences among depths or treatments (Figure 3f).

### 2.4. Abundances of Carbon Cycling and Nitrification Genes

**Figure 4:**
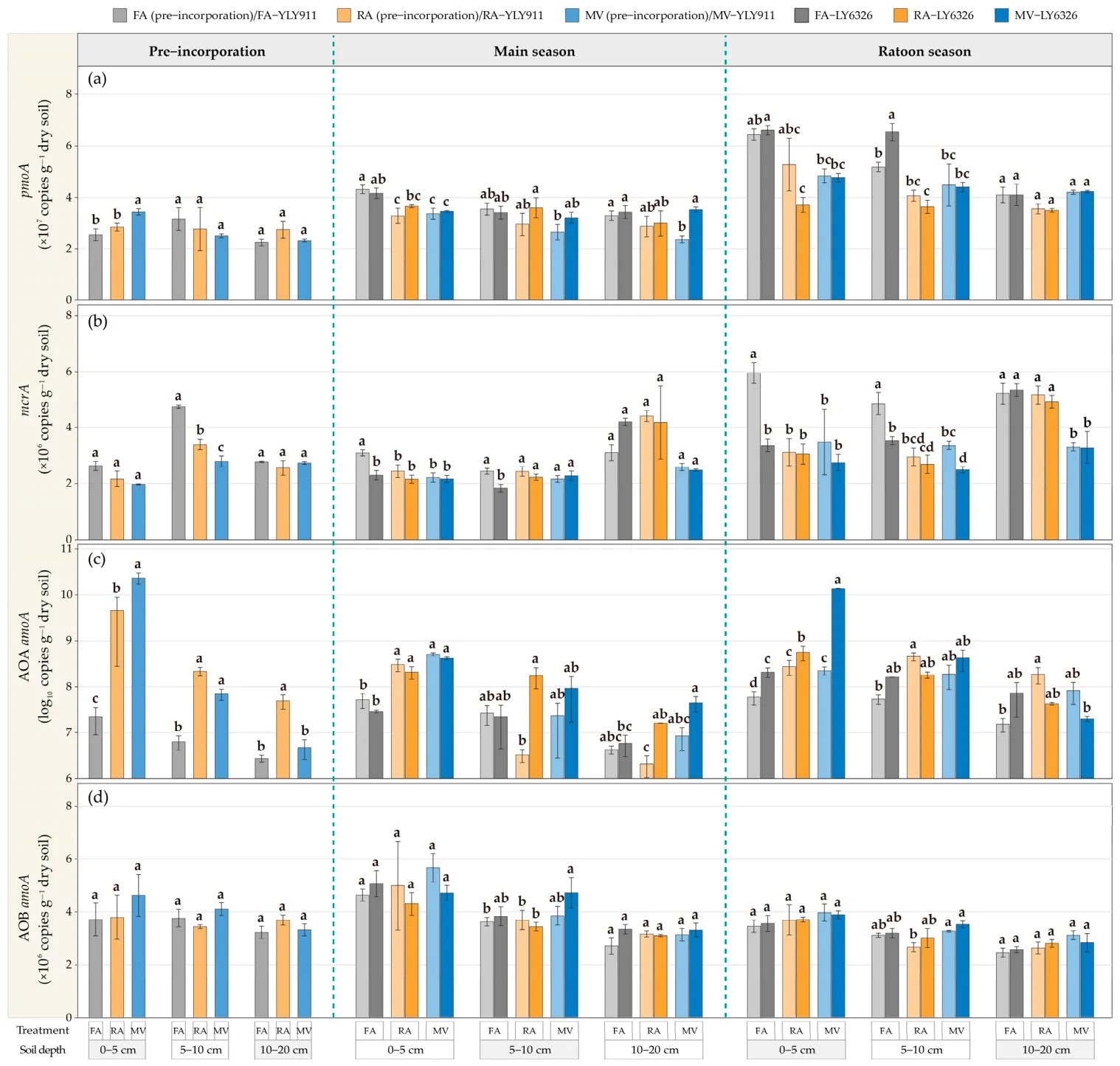
Abundances of (**a**) *pmoA*, (**b**) *mcrA*, (**c**) AOA *amoA* and (**d**) AOB *amoA* genes in soil at three depths under three cropping systems at three sampling stages. Treatment and variety abbreviations are defined in Figure 1. *pmoA*, particulate methane monooxygenase gene (methane oxidation); *mcrA*, methyl-coenzyme M reductase gene (methanogenesis); AOA *amoA* and AOB *amoA*, ammonia monooxygenase genes of ammonia-oxidizing archaea (AOA) and ammonia-oxidizing bacteria (AOB) (ammonia oxidation). Soil samples were collected before residue incorporation and at maturity of the main season and the ratoon season; at pre-incorporation, bars represent the three cropping systems only, whereas in the main and ratoon seasons, they represent the combinations of the three cropping systems and the two ratoon rice varieties. Data are presented as mean ± SE. Within each panel, different lowercase letters indicate significant differences among treatments at the same sampling stage and soil depth at *p* < 0.05 according to the LSD test. The vertical green dashed lines separate the three sampling stages.

The abundances of *pmoA*, *mcrA*, AOA *amoA* and AOB *amoA* genes under the three cropping systems are shown in Figure 4. Green manure significantly affected all four genes, with the strongest effect on *pmoA* and the weakest on AOB *amoA*, whereas rice variety significantly affected *mcrA* and AOA *amoA* but not *pmoA* or AOB *amoA* (Table S4). Significant season × green manure and depth × green manure interactions were detected for *pmoA* and *mcrA*; AOA *amoA* showed a significant depth × green manure interaction together with several higher-order interactions involving rice variety, whereas no interaction was significant for AOB *amoA* (Table S4).

In contrast to the responses of the soil carbon and nitrogen pools, both *pmoA* and *mcrA* were most abundant under FA. Mean *pmoA* abundance was 4.38 × 10^7^, 3.50 × 10^7^ and 3.74 × 10^7^ copies g^−1^ dry soil under FA, RA and MV, with RA and MV being 20.00% and 14.53% lower than FA (Figure 4a). Before residue incorporation, *pmoA* in the 0–5 cm layer was 3.44 × 10^7^ copies g^−1^ under MV, significantly higher than under FA (2.55 × 10^7^) and RA (2.84 × 10^7^) (Figure 4a). This ranking was reversed once rice was established: FA–YLY911 reached 4.31 × 10^7^ copies g^−1^ in the 0–5 cm layer of the main season, significantly higher than RA–YLY911 (3.28 × 10^7^) and both combinations under MV (3.37 × 10^7^ and 3.46 × 10^7^), and FA–LY6326 reached 6.60 × 10^7^ copies g^−1^ in the same layer of the ratoon season, 77.94% higher than the 3.71 × 10^7^ recorded for RA–LY6326 (Figure 4a). *pmoA* abundance increased by 39.10% from 3.34 × 10^7^ in the main season to 4.65 × 10^7^ copies g^−1^ in the ratoon season and decreased with depth from 4.49 × 10^7^ to 3.51 × 10^7^ copies g^−1^ (Figure 4a). Mean *mcrA* abundance was 3.94 × 10^6^, 3.65 × 10^6^ and 2.76 × 10^6^ copies g^−1^ under FA, RA and MV, with MV 29.99% lower than FA (Figure 4b). Treatments already differed significantly in the 5–10 cm layer before residue incorporation, with 4.75 × 10^6^, 3.39 × 10^6^ and 2.78 × 10^6^ copies g^−1^ under FA, RA and MV, and in the 10–20 cm layer of the ratoon season both combinations under MV (3.31 × 10^6^ and 3.28 × 10^6^ copies g^−1^) were significantly lower than the four combinations under FA and RA (4.92 × 10^6^ to 5.34 × 10^6^) (Figure 4b). Unlike *pmoA*, *mcrA* was most abundant at 10–20 cm (4.01 × 10^6^ copies g^−1^), 33.73% higher than at 0–5 cm, and abundance under YLY911 (3.46 × 10^6^ copies g^−1^) was 12.85% higher than under LY6326 (Figure 4b).

The two nitrification genes responded differently. Mean AOA *amoA* abundance was 7.30, 7.72 and 7.85 log_10_ copies g^−1^ dry soil under FA, RA and MV, with RA and MV exceeding FA by 0.42 and 0.55 log units (Figure 4c). Treatments differed significantly at all three depths before residue incorporation, decreasing in the order MV (10.34), RA (8.97) and FA (7.19) in the 0–5 cm layer, with RA and MV (8.32 and 7.80) significantly exceeding FA (6.73) at 5–10 cm and RA being the highest (7.63) at 10–20 cm (Figure 4c). In the 0–5 cm layer of the main season, the four combinations under RA and MV (8.28 to 8.70) were all significantly higher than the two under FA (7.46 and 7.65), and differentiation was most pronounced in the same layer of the ratoon season, in which MV–LY6326 reached 10.13 log_10_ copies g^−1^, significantly exceeding the other five combinations (7.72 to 8.70) (Figure 4c). AOA *amoA* increased by 0.74 log units from 7.40 in the main season to 8.14 log_10_ copies g^−1^ in the ratoon season and decreased with depth from 8.39 to 7.20 log_10_ copies g^−1^, and abundance under LY6326 (7.94 log_10_ copies g^−1^) was 0.34 log units higher than under YLY911 (Figure 4c). AOB *amoA* showed the smallest treatment differences, with a mean of 3.65 × 10^6^ copies g^−1^ under MV, 10.89% higher than the 3.29 × 10^6^ under FA, whereas RA and FA were comparable (Figure 4d). Significant differences were confined to the 5–10 cm layer in both seasons, where MV–LY6326 reached 4.72 × 10^6^ copies g^−1^ in the main season, significantly exceeding FA–YLY911 (3.63 × 10^6^) and both combinations under RA (3.44 × 10^6^ and 3.69 × 10^6^) (Figure 4d). AOB *amoA* declined by 19.41% from 3.96 × 10^6^ in the main season to 3.19 × 10^6^ copies g^−1^ in the ratoon season and decreased with depth from 4.30 × 10^6^ to 2.93 × 10^6^ copies g^−1^ (Figure 4d).

### 2.5. Abundances of Denitrification Genes

**Figure 5:**
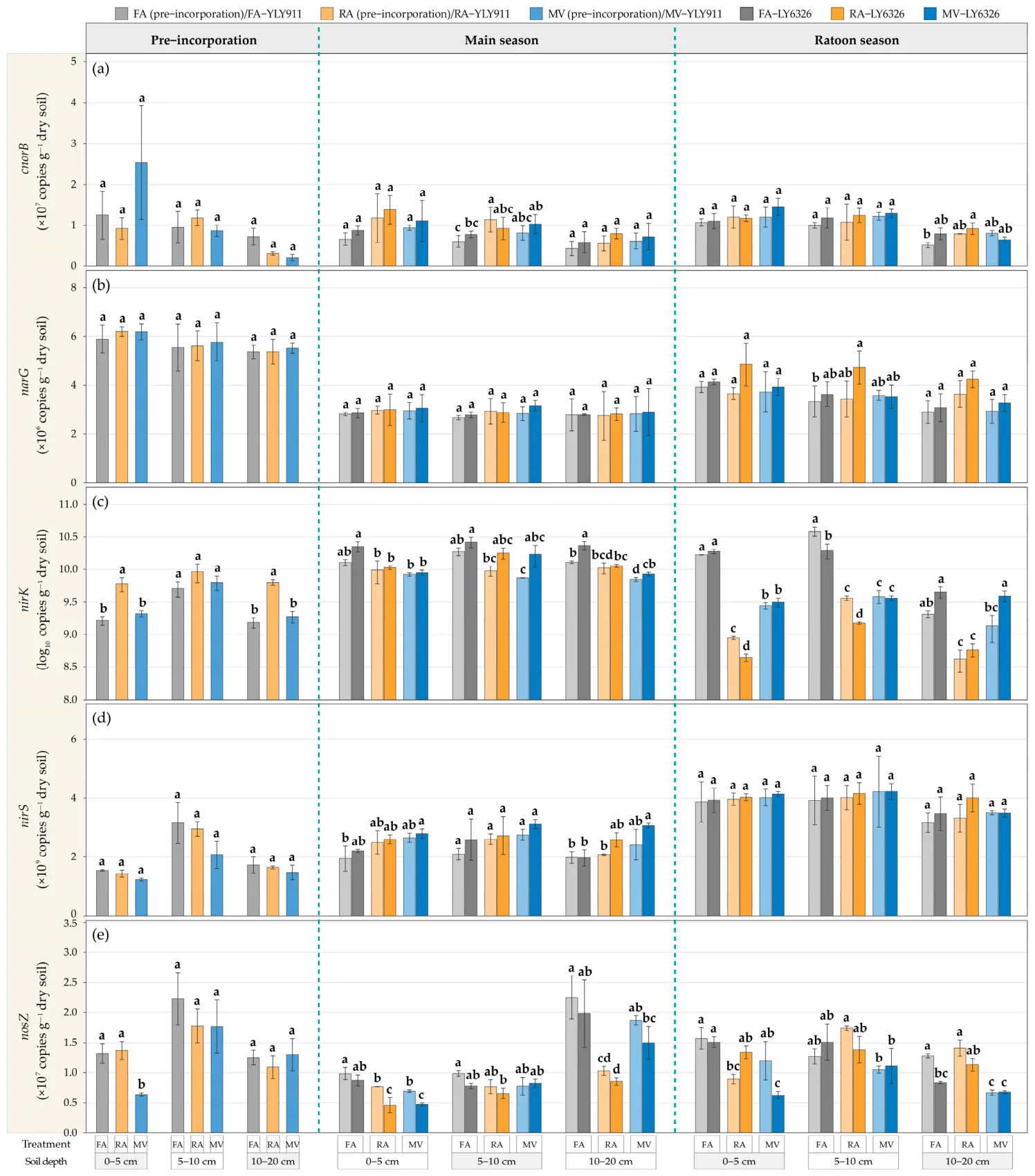
Abundances of (**a**) *cnorB*, (**b**) *narG*, (**c**) *nirK*, (**d**) *nirS* and (**e**) *nosZ* genes in soil at three depths under three cropping systems at three sampling stages. Treatment and variety abbreviations are defined in Figure 1. *cnorB*, nitric oxide reductase gene (denitrification); *narG*, nitrate reductase gene (nitrate reduction); *nirK* and *nirS*, nitrite reductase genes (denitrification); *nosZ*, nitrous oxide reductase gene (denitrification). Soil samples were collected before residue incorporation and at maturity of the main season and the ratoon season; at pre-incorporation, bars represent the three cropping systems only, whereas in the main and ratoon seasons they represent the combinations of the three cropping systems and the two ratoon rice varieties. Data are presented as mean ± SE. Within each panel, different lowercase letters indicate significant differences among treatments at the same sampling stage and soil depth at *p* < 0.05 according to the LSD test. The vertical green dashed lines separate the three sampling stages.

The abundances of *cnorB*, *narG*, *nirK*, *nirS* and *nosZ* genes under the cropping systems are shown in Figure 5. Green manure significantly affected *cnorB*, *nirK*, *nirS* and *nosZ*, with a far stronger effect on *nirK* than on the others, whereas *narG* was the only functional gene in this study affected by neither green manure nor soil depth and showed only a significant seasonal effect; rice variety significantly affected *nirK* and *nosZ* (Table S5). Most interactions were significant for *nirK*, the season × depth interaction for *nosZ* was stronger than either of its main effects, and no interaction was significant for *cnorB*, *narG* or *nirS* (Table S5). Green manure incorporation was associated with higher abundances of *cnorB* and *nirS* but lower abundances of *nirK* and *nosZ* (Figure 5).

Mean *cnorB* abundance was 7.47 × 10^6^, 9.65 × 10^6^ and 9.17 × 10^6^ copies g^−1^ dry soil under FA, RA and MV, with RA and MV 29.22% and 22.67% higher than FA; significant differences were confined to the 5–10 cm layer of the main season and the 10–20 cm layer of the ratoon season (Figure 5a). Mean *narG* abundance was 3.07 × 10^6^, 3.46 × 10^6^ and 3.17 × 10^6^ copies g^−1^ under FA, RA and MV, and treatments differed only in the 5–10 cm layer of the ratoon season (Figure 5b). Mean nirK abundance was 10.08, 9.45 and 9.67 log_10_ copies g^−1^ dry soil under FA, RA and MV, with RA and MV 0.63 and 0.41 log units lower than FA (Figure 5c). Before residue incorporation, RA was significantly higher than FA and MV in the 0–5 and 10–20 cm layers (9.75 and 9.80 versus 9.20–9.31 and 9.17–9.26), whereas this ranking was reversed once rice was established: FA gave the highest abundance at all three depths in the main season, and differentiation was most pronounced in the ratoon season, in which the 0–5 cm layer decreased in the order FA (10.22 and 10.28), MV (9.44 and 9.49) and RA (8.95 and 8.64), FA exceeding RA by 1.46 log units (Figure 5c). nirK declined from 10.08 in the main season to 9.47 log_10_ copies g^−1^ in the ratoon season and decreased with depth from 9.97 to 9.59 log_10_ copies g^−1^, and abundance under LY6326 (9.82 log_10_ copies g^−1^) was 0.09 log units higher than under YLY911 (Figure 5c).

Mean *nirS* abundance was 2.86 × 10^9^, 3.16 × 10^9^ and 3.31 × 10^9^ copies g^−1^ under FA, RA and MV, with MV 15.65% higher than FA and significant differences detected only in the 0–5 and 10–20 cm layers of the main season (Figure 5d). Mean *nosZ* abundance was 1.39 × 10^7^, 1.05 × 10^7^ and 1.01 × 10^7^ copies g^−1^ under FA, RA and MV, with RA and MV 24.00% and 26.94% lower than FA (Figure 5e). In the 10–20 cm layer of the main season, FA–YLY911 reached 2.25 × 10^7^ copies g^−1^, significantly higher than both combinations under RA (1.03 × 10^7^ and 0.86 × 10^7^), whereas in the same layer of the ratoon season the two combinations under MV were the lowest (0.67 × 10^7^ and 0.68 × 10^7^) (Figure 5e). The vertical distribution of *nosZ* differed between seasons, being highest at 10–20 cm in the main season (1.58 × 10^7^ copies g^−1^) but at 5–10 cm in the ratoon season (1.34 × 10^7^ copies g^−1^), and abundance under YLY911 (1.18 × 10^7^ copies g^−1^) was 14.46% higher than under LY6326 (Figure 5e). Among seasonal changes, *nirS* showed the largest increase, rising by 55.13% from 2.49 × 10^9^ in the main season to 3.86 × 10^9^ copies g^−1^ in the ratoon season, followed by *narG* and *cnorB* (28.68% and 23.06%), whereas *nirK* was the only gene of the five that declined in the ratoon season (Figure 5a–d).

### 2.6. Relationships Between Soil Properties, Functional Gene Abundances and Theoretical Grain Yield

**Figure 6:**
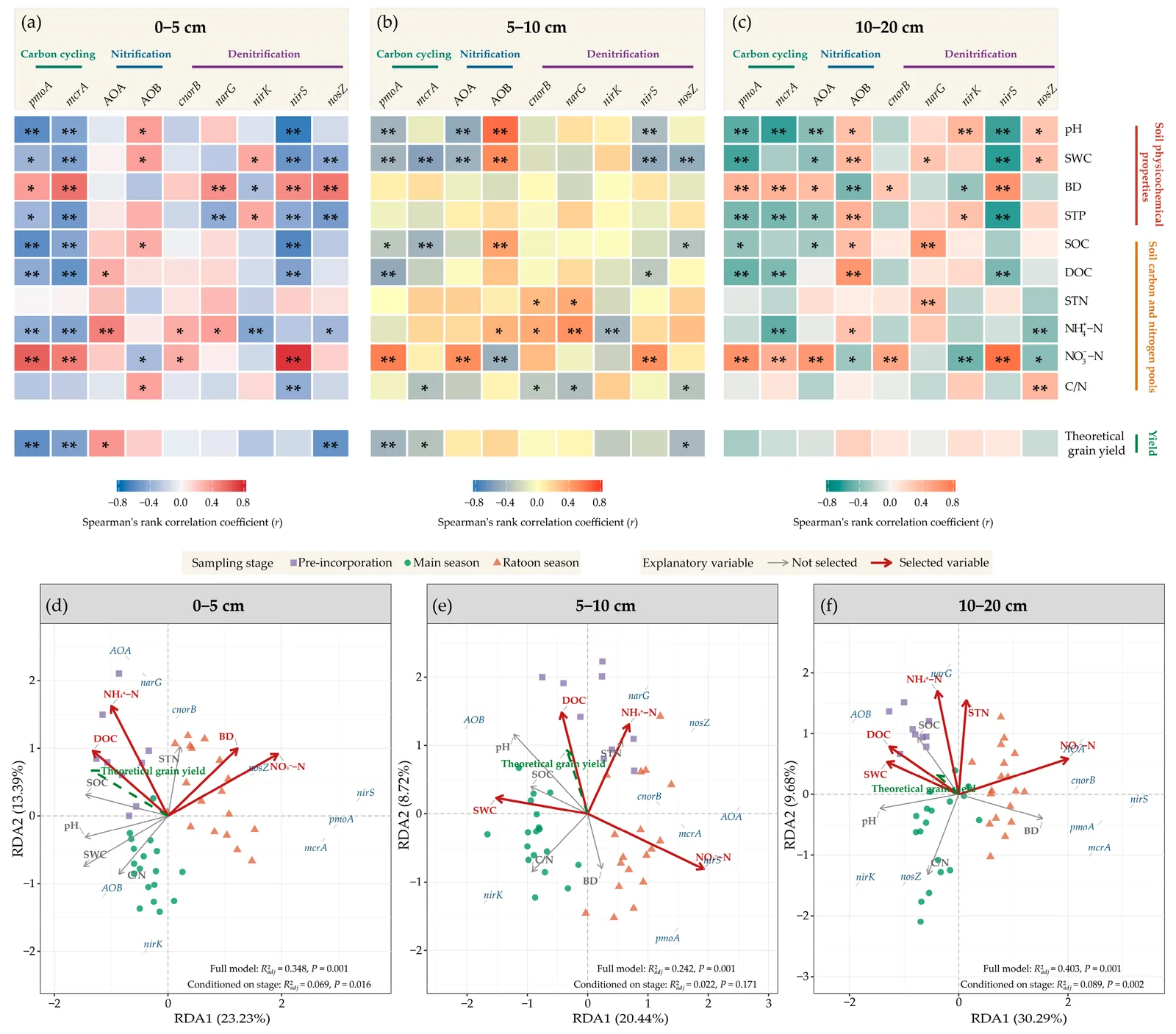
Relationships between soil properties, functional gene abundances and theoretical grain yield at three soil depths: (**a**–**c**) Spearman’s rank correlation analysis at 0–5, 5–10 and 10–20 cm soil depth; (**d**–**f**) redundancy analysis at the corresponding soil depths. Functional genes are defined in Figure 4 and Figure 5. SWC, soil water content; BD, soil bulk density; STP, soil total porosity; SOC, total soil organic carbon; DOC, soil dissolved organic carbon; STN, soil total nitrogen; NH_4_^+^–N, soil ammonium nitrogen; NO_3_^−^–N, soil nitrate nitrogen; C/N, carbon to nitrogen ratio. Gene abundances were log_10_-transformed before both analyses. In (**a**–**c**), asterisks denote significant correlations, * 0.01 ≤ *p* < 0.05 and ** *p* < 0.01; correlations with soil variables are based on samples from the three sampling stages, *n* = 45, and those with theoretical grain yield on samples from the main and ratoon seasons, *n* = 36, as no yield was available before residue incorporation. In (**d**–**f**), redundancy analysis was performed on standardized gene abundances constrained by the standardized soil variables shown, *n* = 45; STP was excluded because of its collinearity with BD, as shown in (**a**–**c**). Forward selection was used to identify significant explanatory variables, indicated by red arrows, whereas the ordination was based on the full model containing all variables shown. Theoretical grain yield, dashed green arrow, was fitted passively onto the ordination, *n* = 36. *p* values are based on permutation tests with 999 permutations.

**Figure 7:**
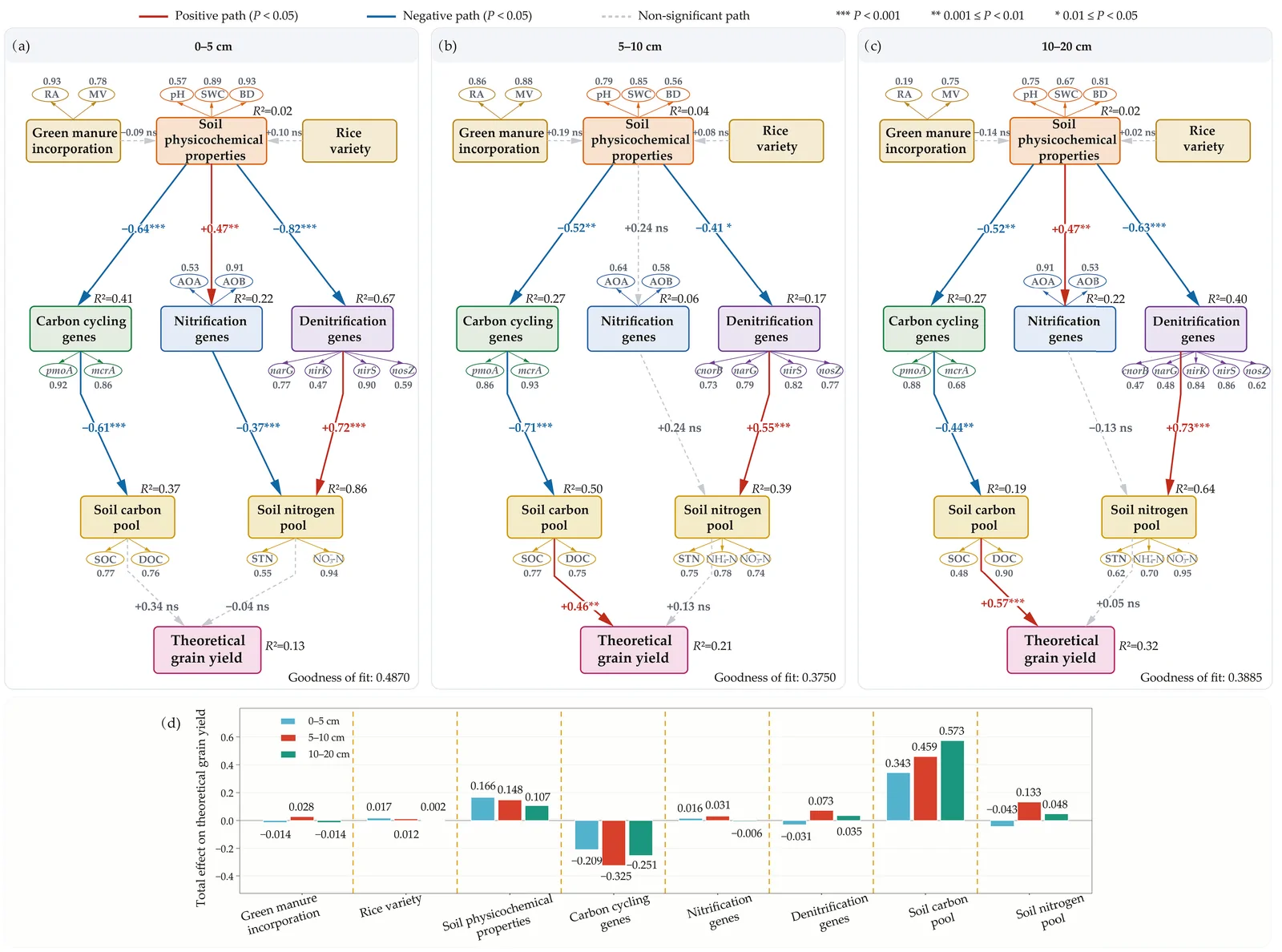
Partial least squares path modelling of the effects of green manure incorporation, rice variety, soil physicochemical properties and functional gene abundances on theoretical grain yield at three soil depths: (**a**–**c**) path models at 0–5, 5–10 and 10–20 cm soil depth; (**d**) standardized total effects on theoretical grain yield at the three soil depths. Treatment and variety abbreviations are defined in Figure 1; functional genes are defined in Figure 4 and Figure 5. SWC, soil water content; BD, soil bulk density; SOC, total soil organic carbon; DOC, soil dissolved organic carbon; STN, soil total nitrogen; NH_4_^+^–N, soil ammonium nitrogen; NO_3_^−^–N, soil nitrate nitrogen. In (**a**–**c**), rectangles denote latent variables and ellipses the observed variables used as their indicators, with outer loadings given beside each indicator; numbers on the arrows are standardized path coefficients and *R*^2^ denotes the variance of each endogenous latent variable explained by the model.

The relationships between soil properties, functional gene abundances and theoretical grain yield at the three soil depths are shown in Figure 6, and the path analysis of their effects on theoretical grain yield in Figure 7. Spearman’s rank correlation analysis showed that the correlation patterns differed among soil depths and were most numerous in the 10–20 cm layer (Figure 6a–c). Soil pH and SWC were negatively correlated with most functional genes, whereas BD was positively correlated with them (Figure 6a–c). *pmoA* was highly significantly negatively correlated with pH at all three depths (*r* = −0.60, −0.44 and −0.51) and positively correlated with NO_3_^−^–N (*r* = 0.61, 0.55 and 0.64), and *mcrA* showed a similar pattern (Figure 6a–c). The positive correlation between *nirS* and NO_3_^−^–N was the strongest at all three depths (*r* = 0.75, 0.48 and 0.83) (Figure 6a–c). Significant correlations between theoretical grain yield and functional genes occurred only in the 0–5 and 5–10 cm layers, where yield was significantly negatively correlated with *pmoA* (*r* = −0.58 and −0.44), *mcrA* (*r* = −0.48 and −0.35) and *nosZ* (*r* = −0.56 and −0.42), whereas no correlation was significant in the 10–20 cm layer (Figure 6a–c). BD and STP were correlated with the genes with opposite signs but similar magnitudes and were thus highly collinear, so STP was excluded from the subsequent redundancy analysis (Figure 6a–c).

In the redundancy analysis, the full models were highly significant at all three depths (*p* = 0.001), with the first two axes explaining 29.16% to 39.97% of the variation and adjusted coefficients of determination of 0.348, 0.242 and 0.403, being highest in the 10–20 cm layer (Figure 6d–f). When conditioned on sampling stage, the models remained significant in the 0–5 and 10–20 cm layers (*R*^2^adj = 0.069 and 0.089; *p* = 0.016 and 0.002) but not in the 5–10 cm layer (*R*^2^adj = 0.022, *p* = 0.171), indicating that the gene–environment associations in the middle layer were driven mainly by seasonal variation (Figure 6d–f). Forward selection retained NH_4_^+^–N, DOC and NO_3_^−^–N at all three depths, SWC in the 5–10 and 10–20 cm layers, BD only in the 0–5 cm layer and STN only in the 10–20 cm layer (Figure 6d–f).

Partial least squares path modelling revealed a consistent structure across the three soil depths (Figure 7a–c). Soil physicochemical properties had significant negative paths to both carbon cycling genes (path coefficients −0.52 to −0.64) and denitrification genes (−0.41 to −0.82); carbon cycling genes had significant negative paths to the soil carbon pool (−0.44 to −0.71), and denitrification genes had significant positive paths to the soil nitrogen pool (0.55 to 0.73) (Figure 7a–c). The path coefficient from the soil carbon pool to theoretical grain yield increased with soil depth from 0.34 to 0.57 and was significant in the 5–10 and 10–20 cm layers, with the variance explained by the model rising correspondingly from 0.13 to 0.32, whereas the path from the soil nitrogen pool was not significant at any depth (Figure 7a–c). The direct paths from green manure incorporation and rice variety to soil physicochemical properties were not significant at any depth (Figure 7a–c). Analysis of standardized total effects showed that the soil carbon pool had the largest positive total effect on theoretical grain yield at all three depths, increasing with depth from 0.343 to 0.573, followed by carbon cycling genes as the largest negative effect (−0.209 to −0.325), whereas the absolute total effects of green manure incorporation and rice variety did not exceed 0.03 at any depth (Figure 7d).

## 3. Discussion

### 3.1. Soil Carbon and Nitrogen Availability

Residue incorporation raised DOC well before it altered SOC (Figure 3a,b). DOC, the most labile fraction of soil organic carbon, was elevated under both residues and rose most sharply in the surface soil before incorporation, where rapeseed reached 884.87 mg kg^−1^ against 450.80 mg kg^−1^ under fallow, and it remained higher than fallow in the deep layer of both rice seasons (Figure 3b). Incubation studies of paddy soils report a similarly rapid release of dissolved organic carbon following residue addition [23]. SOC, in contrast, increased only marginally, differing from fallow only in the surface and middle layers of the ratoon season, where milk vetch reached 13.37 g kg^−1^ at 5–10 cm against 12.68 g kg^−1^ under fallow (Figure 3a). Such a pronounced DOC response alongside a muted SOC response is expected within a single cycle, since green manure consists largely of labile compounds, whereas an appreciable build-up of stable organic carbon typically requires years of repeated incorporation rather than one season [24,25]. The residues thus provided a readily soluble carbon source well before the more slowly forming SOC pool could register the input.

**Figure 8:**
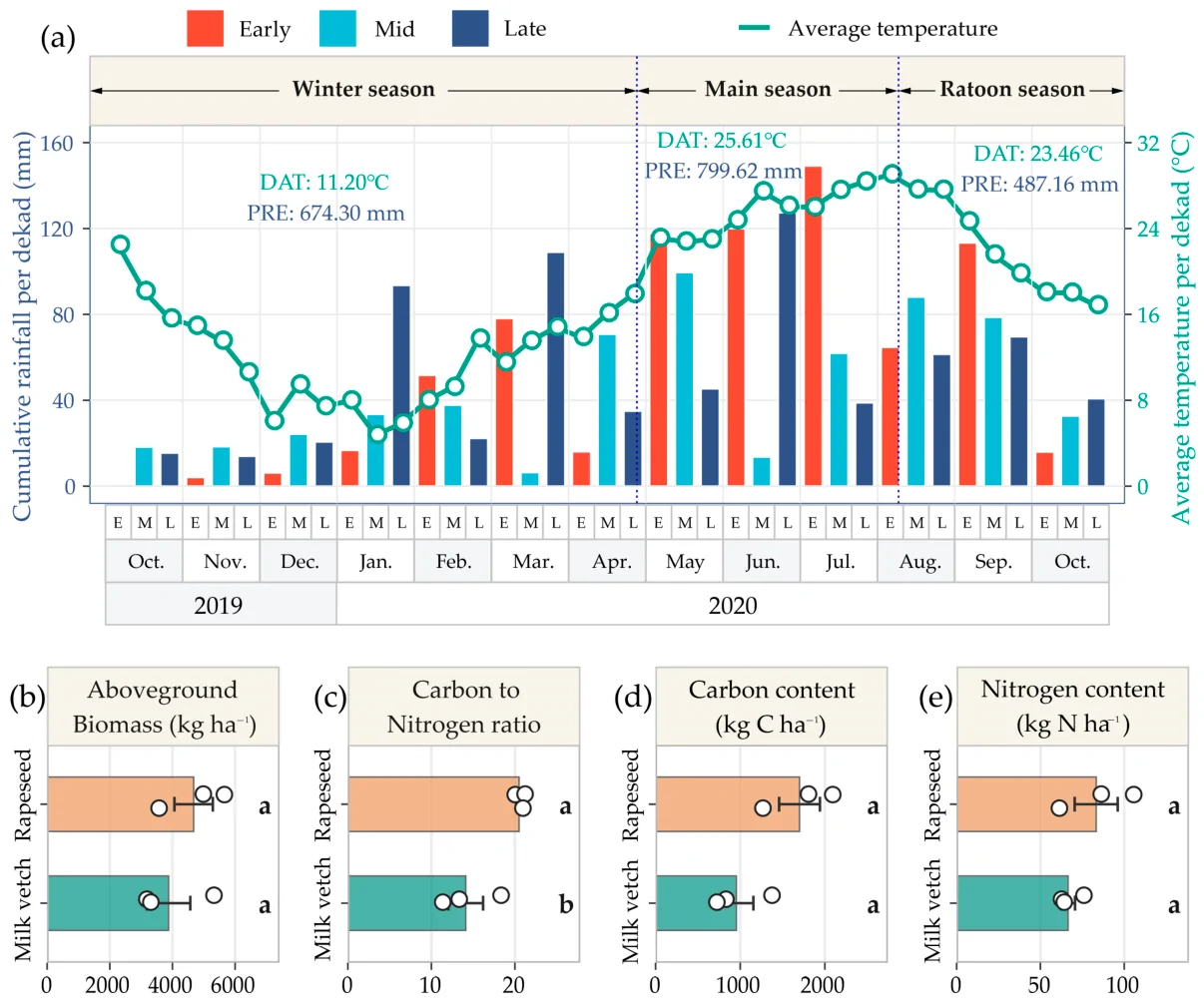
Climatic conditions during the experimental period and the biomass and carbon–nitrogen characteristics of the two green manure residues: (**a**) cumulative rainfall and average air temperature per dekad from October 2019 to October 2020, with the seasonal mean air temperature (DAT) and cumulative precipitation (PRE) of the winter, main and ratoon seasons indicated; (**b**–**e**) aboveground biomass, carbon to nitrogen ratio, carbon content and nitrogen content of rapeseed and milk vetch. In (**a**), bars show the rainfall of the early (E), middle (M) and late (L) periods of each month and the line shows the average temperature per dekad. In (**b**–**e**), circles denote individual replicates, and different lowercase letters indicate significant differences between the two green manure crops at *p* < 0.05.

The nitrogen response was dominated by NH_4_^+^-N and differed sharply between the two residues (Figure 3d). NH_4_^+^-N was the pool most strongly affected, and its response depended on both season and depth, consistent with the significant season × treatment, depth × treatment and three-way interactions (Table S3). Milk vetch already carried the highest surface NH_4_^+^-N before incorporation (73.87 mg kg^−1^), and in the main season the residues raised NH_4_^+^-N above fallow mainly in the surface soil (Figure 3d). The sharpest divergence emerged in the ratoon season, where milk vetch exceeded fallow at all three depths while rapeseed did not, so that milk vetch was also significantly higher than rapeseed throughout the profile (Figure 3d). The two residues produced similar aboveground biomass (Figure 8b), so this contrast reflects their chemistry rather than the amount returned [26]. Their C/N ratios differed significantly in the present study, at 14.12 for milk vetch against 20.50 for rapeseed (Figure 8c). As a legume with a low C/N ratio, milk vetch decomposes rapidly and releases mineral nitrogen, and has been widely reported to supply enough nitrogen to replace part of the mineral fertiliser in southern rice systems [27,28], whereas the wider C/N ratio of rapeseed favours microbial immobilisation and yields comparatively little available nitrogen [13,29]. The surface NH_4_^+^-N maximum under milk vetch occurred in the ratoon season rather than immediately after incorporation, suggesting that part of the released nitrogen was retained in the soil and re-mineralised over the year rather than being nitrified or taken up during the main season [30]. NO_3_^−^-N, in contrast, was governed mainly by season, increasing several-fold from the main to the ratoon season with only a minor treatment effect in the deeper layers (Figure 3e). This predominance of NH_4_^+^-N over NO_3_^−^-N is consistent with a low-pH paddy in which residue-derived nitrogen accumulated chiefly as ammonium and only a small fraction was nitrified [31]. Against these pronounced shifts in mineral nitrogen, STN and the soil C/N ratio changed little, STN differing from fallow only in the deep layer of the ratoon season and C/N varying only with season, falling from 14.75 to 13.48 (Figure 3c,f). This indicates that the added carbon and nitrogen entered these large, slowly cycling pools without altering their size or proportion within a single cycle. Notably, the carbon and nitrogen returned by rapeseed were numerically even slightly higher than those of milk vetch, though the difference was not significant (Figure 8d,e); the fact that rapeseed nonetheless supplied less available nitrogen than milk vetch reinforces that the nitrogen release was governed by residue chemistry, in particular the C/N ratio, rather than by the quantity of carbon and nitrogen returned.

### 3.2. Carbon-Cycling Genes

The two carbon-cycling genes did not respond to residue in a single direction, and their response varied with season and depth (Figure 4a,b; Table S4). When the two rice seasons were considered together, *pmoA* and *mcrA* appeared more abundant under fallow (Figure 4a,b), but this overall impression was not consistent across stages or depths. In the surface soil of both rice seasons, *pmoA* was highest under fallow, whereas before incorporation, it had been significantly higher under milk vetch, and at 10–20 cm the treatments converged (Figure 4a). The significant season × treatment and depth × treatment interactions confirm that the effect of residue on *pmoA* was conditional rather than uniform (Table S4). This surface pattern is better interpreted through the substrate relations of methanotrophs than as a direct effect of residue on the gene [32]. Across the three depths, *pmoA* was negatively correlated with pH and positively correlated with NO_3_^−^-N (Figure 6a–c), a pattern reported for paddy methanotrophs, whose abundance and activity tend to decline as pH rises and to increase with nitrogen availability [33], and methane oxidation is further inhibited when ammonium accumulates to high concentrations [34]. The highest NH_4_^+^-N, recorded under milk vetch in the ratoon-season surface soil, coincided with the lowest *pmoA* there; this co-occurrence is consistent with such inhibition, although the present data cannot establish it as the cause.

The methanogenic gene *mcrA* showed a partly similar and partly distinct pattern (Figure 4b). Like *pmoA*, it was most abundant under fallow in the surface soil (Figure 4b), but unlike *pmoA* it peaked in the deepest layer rather than at the surface, and it was the only carbon-cycling gene significantly affected by variety (Table S4). The deeper maximum is consistent with the more reduced conditions expected at depth in a flooded paddy, which favour the strictly anaerobic methanogens, whereas the aerobic methanotrophs carrying *pmoA* were more abundant nearer the surface [35]. A comparable modulation of methane-related genes by milk vetch was reported by Li et al. [36], who found that combining Chinese milk vetch with nitrogen reduction reshaped carbon-cycling functional genes, including the methanogenesis gene *mcrA*, in a paddy soil, supporting our finding that green manure incorporation altered the methanogenic and methanotrophic gene abundances rather than leaving them unchanged. It should be noted that the *pmoA* and *mcrA* values reported here are gene copy numbers quantified by quantitative polymerase chain reaction (qPCR). They represent the genetic potential for methane oxidation and methanogenesis rather than the actual process rates, as neither methane flux nor redox potential was measured in this study, and the abundance changes should therefore be interpreted as shifts in functional potential rather than as changes in the processes themselves.

### 3.3. Nitrogen-Cycling Genes

The nitrogen-cycling genes ranged from unresponsive to sharply divergent, and the two ammonia oxidisers separated most clearly (Figure 4c,d; Figure 5). AOA amoA increased under both residues, most strongly under milk vetch in the ratoon-season surface soil, whereas AOB amoA changed little and showed no significant interactions (Figure 4c,d; Table S4). This separation is consistent with the acidic condition of the soil. Ammonia-oxidising archaea are repeatedly found to dominate nitrification in acidic soils, and to be favoured where ammonia is supplied slowly through mineralisation rather than as a concentrated fertiliser pulse [37,38]. This dominance of ammonia-oxidising archaea is in line with Wang et al. [39], who reported that AOA amoA was more abundant than AOB amoA throughout three Chinese paddy soil profiles, confirming that archaea were the principal ammonia oxidisers in these acidic paddies as in our soil. The parallel rise of AOA amoA and NH_4_^+^-N under milk vetch is therefore expected (Figure 3d; Figure 4c), while AOB amoA, whose members are better suited to higher ammonia and pH, remained largely unchanged.

The denitrification genes were similarly uneven (Figure 5). *narG* was the least responsive gene measured, unaffected by treatment or depth and varying only with season (Figure 5b; Table S5), even though its substrate NO_3_^−^-N rose several-fold from the main to the ratoon season (Figure 3e). The nitrate-reduction step indexed by *narG* thus appeared decoupled from the residue-driven supply of nitrate. *nirK* and *nirS* catalyse the same reaction but mark two ecologically distinct denitrifier groups [40], and here they moved in opposite directions under residue, *nirK* declining while *nirS* rose (Figure 5c,d). This divergence agrees with the view that the two groups occupy different niches and respond differently, at times oppositely, to organic inputs and to carbon and nitrogen gradients [41,42,43]. Along the NO_3_^−^-N gradient of this study, which rose several-fold from the main to the ratoon season and declined with depth, the two genes responded in opposite directions (Figure 3e and Figure 5c,d). *nirS* correlated positively with NO_3_^−^-N at all three depths and rose with it into the ratoon season (Figure 6a–c), consistent with reports that *nirS*-type denitrifiers depend closely on nitrate availability [44]. *nirK*, in contrast, correlated negatively with NO_3_^−^-N, most clearly in the 10–20 cm layer (Figure 6c), and declined most in the ratoon-season surface soil, where NO_3_^−^-N was highest and *nirK* under the green manure treatments fell well below that under fallow (Figure 5c). This opposite alignment with the measured nitrate gradient reflects the ecological differentiation and contrasting nitrate dependence of the two denitrifier groups. A comparable depth-related separation of the two nitrite reducers was reported by Wang et al. [39], who found *nirS* and *nirK* to be dominant in topsoil and deeper soil, respectively, along Chinese paddy profiles, indicating that the two genes occupy distinct niches. Khanal and Lee [45] likewise quantified *nirK*, *nirS* and other nitrogen-cycling genes in a paddy soil by qPCR, confirming that these denitrification genes are consistently detectable and differentially distributed in paddy systems, which supports our observation that *nirK* and *nirS* responded distinctly rather than in unison. Of all the denitrification genes, *nirK* showed the strongest response to residue (Table S5), in keeping with reports that *nirK*-type denitrifiers are especially sensitive to nitrogen inputs [41]. *nosZ*, which catalyses the terminal step, was lower under residue and shifted its depth distribution between seasons (Figure 5e). That the nitrite- and nitrous-oxide-reductase genes shifted while *narG* did not indicates that residue incorporation was associated with particular steps of the denitrification pathway rather than with the pathway as a whole. It should be noted, however, that the values reported here are gene copy numbers quantified by qPCR. They represent the genetic potential of the nitrifying and denitrifying groups rather than their actual activity, and cannot be equated with the real process rates of ammonia oxidation, denitrification or nitrous oxide emission, none of which were measured in this study. The abundance changes described above should therefore be interpreted as shifts in functional potential.

### 3.4. Linkages Among Soil Properties, Functional Genes and Grain Yield

The correlation, ordination and path analyses pointed to the same small set of labile pools as the variables most closely associated with functional gene abundance and, through it, with yield. In the redundancy analysis, forward selection retained NH_4_^+^-N, DOC and NO_3_^−^-N at all three depths (Figure 6d–f), so these pools, rather than the physical properties of the soil, were the principal correlates of gene abundance. At 5–10 cm the constrained model lost significance once sampling stage was partialled out (Figure 6e), indicating that the gene–environment association in the middle layer was largely seasonal. The path model then arranged the variables in sequence, linking soil properties to the functional genes, the genes to the soil carbon and nitrogen pools, and the carbon pool to theoretical grain yield, with the last association strengthening with depth (Figure 7a–c). The direct paths from residue treatment and variety to yield were not significant, and the carbon pool carried the largest positive total effect on yield (Figure 7d). Read together with the higher yield under both residues, which widened into the ratoon season where fallow gave the lowest value (Figure 1), this is consistent with winter treatment being associated with yield through the soil rather than acting on it directly, the indirect pattern anticipated in the third hypothesis. The first hypothesis was only partly supported, since yield rose under residue as expected, but the topsoil physicochemical properties did not improve as a general effect, which places the residue effect in the carbon and nitrogen pools instead. Variety had a limited role, reaching only *mcrA* and AOA *amoA* among the measured variables, with no residue × variety interaction for any variable (Tables S4 and S5), so the direction and size of the residue effect were much the same across the two cultivars.

The main contribution of this study is to have followed residue-driven shifts in carbon- and nitrogen-cycling genes through a full main-plus-ratoon cycle, resolved by season and depth, in a system for which most evidence has come from single-season or double-rice fields. Two features stand out. Several genes reversed direction between the pre-incorporation stage and the rice seasons, and between the surface and deep soil, so that treatment means pooled over the profile can misrepresent the direction of change. In addition, *narG* remained constant while the nitrite- and nitrous-oxide-reductase genes shifted, which suggests that winter management was associated with selected steps of denitrification rather than with the whole pathway. For practice, the consistency of the residue effect across both cultivars suggests that winter green manuring need not be matched to a particular variety in this system. No inference is drawn about greenhouse-gas outcomes, since no fluxes were measured.

### 3.5. Limitations and Perspectives

The experiment covered one ratoon cycle at a single acidic-paddy site, so the patterns describe the response to one winter’s residue within that cycle rather than a cumulative or long-term trend, and the stability of the physical properties in particular may not persist once residue is returned over successive years, as long-term green manure trials have reported for bulk density and pH [46]. Because the analysis was resolved by season and depth rather than relying on pooled means, the reversals were identified rather than averaged away, and the yield-related conclusions rest on the path structure across three depths rather than on any single value, so the single-cycle scope limits the extrapolation of the findings without undermining them. In the same way, the finding that the green manure effect did not depend on rice variety was established for the two ratoon rice cultivars examined here (YLY911 and LY6326) under a single acidic-paddy site and one cropping cycle, with no significant residue-by-variety interaction for the measured variables. This conclusion therefore applies within the range of cultivars and conditions tested, and whether the variety-independence of the green manure effect holds for other cultivars, soils and climates remains to be confirmed in broader trials.

The microbial groups were characterised only by marker-gene abundance, which indexes genetic potential rather than activity, and neither gas fluxes nor redox conditions were recorded, so the shifts in *pmoA*, *nirK*, *nirS* and *nosZ* cannot yet be linked to methane or nitrous oxide processes. Stable-isotope probing and metatranscriptomics would test whether the abundance shifts correspond to active populations rather than to potential alone. Controlled microcosms would allow the opposing responses of *nirK* and *nirS* to be examined as cause rather than correlation. Paired flux and redox monitoring would connect the functional genes to the greenhouse-gas processes they are presumed to represent. Multi-year, multi-site trials across contrasting soils and climates would establish whether the within-cycle responses accumulate, stabilise or reverse beyond the acidic paddy studied here. Extending the sampling to the roots and rhizosphere of the regenerating tillers would further link the winter residue, the main crop and the ratoon crop through the soil microbial community across the full cycle.

## 4. Materials and Methods

### 4.1. Site Description

The experiment was conducted at the experimental farm of Hunan Agricultural University in Yanxi Town, Liuyang City, Hunan Province, China (28°30′ N, 113°83′ E; 100 m above sea level). The region has a humid subtropical monsoon climate. The average air temperature and cumulative precipitation per dekad during the experimental period, together with the seasonal mean air temperature and cumulative precipitation for the winter, main and ratoon seasons, are shown in Figure 8a. The soil is a red-yellow mud, a clayey paddy soil classified as a Stagnic Anthrosol (Chinese Soil Taxonomy) [47] or a Hydragric Anthrosol (World Reference Base for Soil Resources) [48]. The previous cropping pattern was double-season rice. The main soil properties at the three depths at the beginning of the experiment are given in Table S6.

### 4.2. Experimental Design

A ratoon rice system had been established in April 2019 on a field previously under double-season rice, and the field had at that time been shaped into a bed-furrow layout to restrict the lateral exchange of water and nutrients among plots: raised beds 1.20 m wide were separated by furrows 0.20 m wide and 0.20 m deep, nine main plots (61.60 m^2^ each) were each bounded by a black-plastic-filmed ridge 0.40 m wide and 0.30 m high, and the ditches between plots (0.30 m wide, 0.30 m deep) served for irrigation and drainage. After the ratoon crop of that system was harvested in October 2019, the present experiment was initiated on this existing layout and ran for one complete cycle from October 2019 to October 2020. The treatments were arranged as a split-plot design replicated three times. The cropping system formed the main-plot factor and was randomly allocated to the nine main plots as three treatments, winter fallow–ratoon rice (FA, the control without green manure), rapeseed–ratoon rice (RA) and Chinese milk vetch–ratoon rice (MV); the ratoon rice variety formed the subplot factor, each main plot being split for Yliangyou 911 (YLY911) and Liangyou 6326 (LY6326).

### 4.3. Agronomic Management

Rapeseed (*Brassica napus* L.; Xiangzayou 199) and Chinese milk vetch (*Astragalus sinicus* L.; Xiangzi 4) were broadcast onto the beds of the RA and MV plots on 14 October 2019 at 5.00 and 62.50 kg ha^−1^, respectively, and were grown through the winter without irrigation or fertilisation. A herbicide was applied to the FA plots only once, when the plots were prepared at the beginning of winter. At full bloom, on 23 April 2020, the shoots of both green manure crops were chopped into 5–10 cm segments and incorporated into the upper 0.20 m of soil with a small rotary tiller, about 15 days before rice transplanting.

The ratoon rice was grown in the same way under every treatment. Seedlings of YLY911 and LY6326 were raised from 20 March 2020 and set out by hand on 27 April at two per hill, 13.30 cm apart within rows 30.00 cm apart, giving 250,639 hills ha^−1^. The main crop matured over 142 days and was harvested on 9 August, whereas the ratoon crop occupied a further 61-day period and was taken on 9 October. During the main crop season, nitrogen (46.00% urea) was applied at 200 kg N ha^−1^ in three splits at a basal:tillering:panicle-initiation ratio of 5:2:3, phosphorus (12.00% calcium superphosphate) at 90 kg P_2_O_5_ ha^−1^ entirely as a basal dose, and potassium (60.00% potassium chloride) at 120 kg K_2_O ha^−1^ in two splits at a basal:panicle-initiation ratio of 4:3. Water was managed under an alternate wetting and drying regime, and the main crop was cut at maturity to a stubble height of about 40 cm. During the ratoon crop season, nitrogen was applied at 150 kg N ha^−1^ in two equal splits, the first 10 days after the main crop headed to stimulate axillary buds and the second 10 days after its harvest to drive regenerated tillering. A 3–5 cm water layer was imposed as soon as the main crop was removed, and the furrows were thereafter managed so that the beds stayed moist without standing water until the ratoon crop headed. After the ratoon harvest the straw was cleared without delay, and pests, diseases and weeds were kept in check by conventional means throughout the season.

### 4.4. Soil Sampling

Soil was collected on three occasions across the cropping cycle. The first sampling was carried out on 17 April 2020, immediately before the green manure was incorporated and referred to as the pre-incorporation stage, the second on 12 August 2020 shortly after the main crop was harvested, and the third on 12 October 2020 shortly after the ratoon crop was harvested. On each occasion, five cores were taken at random with a 5 cm auger from each of the 0–5, 5–10 and 10–20 cm layers within every subplot, and the five cores of a given layer were pooled and homogenised into a single composite sample after visible roots and stones had been removed. Each composite sample was divided into three portions. One portion was kept field-moist at 4 °C for the determination of SWC, NH_4_^+^-N, NO_3_^−^-N and DOC, one was stored at −20 °C for deoxyribonucleic acid (DNA) extraction, and one was air-dried and sieved for the determination of pH, SOC and STN. Additional undisturbed cores were collected simultaneously from the same layers with a stainless-steel ring to measure BD.

### 4.5. Soil Physicochemical Analysis

Soil pH was measured with a compound electrode (FE28, Mettler-Toledo, Greifensee, Switzerland) at a soil-to-water ratio of 1:5 [49]. SWC was determined by oven-drying at 105 °C for 48 h. BD was measured by the ring-knife method, and STP was calculated as STP (%) = (1 − BD/soil specific gravity) × 100, with the soil specific gravity taken as 2.65 g cm^−3^ [11]. SOC was determined by potassium dichromate oxidation [50]. DOC was extracted with ultrapure water at a soil-to-water ratio of 1:5, and after shaking, centrifugation and filtration through a 0.45 μm membrane, the DOC in the filtrate was measured by potassium dichromate oxidation [11]. STN was determined by Kjeldahl digestion [51]. NH_4_^+^-N and NO_3_^−^-N were extracted with 2 mol L^−1^ potassium chloride at a soil-to-extractant ratio of 1:5 and determined by the indophenol-blue colorimetric method and the ultraviolet spectrophotometry method, respectively [50]. The soil C/N ratio was calculated as the ratio of SOC to STN.

### 4.6. Functional Gene Quantification

Total soil DNA was extracted from 0.50 g of fresh soil with the FastDNA Spin Kit for Soil (MP Biomedicals, Santa Ana, CA, USA) following the manufacturer’s instructions. The extracted DNA was dissolved in 70 μL of Tris-EDTA buffer, and its concentration and purity were checked with a NanoDrop ND-1000 spectrophotometer (NanoDrop Technologies Inc., Wilmington, DE, USA) before storage at −20 °C.

The abundances of nine functional genes involved in carbon and nitrogen cycling were quantified by qPCR, namely the methane-related genes *pmoA* and *mcrA*, the ammonia monooxygenase genes of ammonia-oxidising archaea (AOA *amoA*) and bacteria (AOB *amoA*), and the denitrification genes *cnorB*, *narG*, *nirK*, *nirS* and *nosZ*. Primer specificity was first confirmed by PCR and agarose gel electrophoresis, in which each gene amplified from the soil DNA produced a single band of the expected length in three independent replicates. Standard curves were constructed for each gene by purifying the corresponding PCR product, ligating it into the pEASY-T1 vector (TransGen Biotech, Beijing, China), transforming it into *Escherichia coli*, and selecting positive white clones by blue-white screening. The inserted fragment was confirmed by sequencing, and the plasmid was quantified and serially diluted tenfold. Amplification efficiencies of 85–105% and R^2^ > 0.99 were obtained for all genes. Each qPCR was run in a 10.00 μL reaction containing 1.00 μL of DNA template, 5.00 μL of SYBR Premix Ex Taq (Perfect Real Time) (Takara Bio, Kusatsu, Shiga, Japan), 0.50 μL of each primer and 3.00 μL of sterile double-distilled water, with sterile water substituted for the template as a negative control. Each sample was quantified in three technical replicates, giving three biological and three technical replicates for each treatment at every depth and sampling stage. All reactions were performed on a LightCycler 480 real-time PCR system (Roche Diagnostics, Basel, Switzerland). The primer pairs and their sources, together with the thermal cycling conditions for each gene, are given in Table S7 [52,53,54,55,56,57].

### 4.7. Plant Sampling and Theoretical Grain Yield

Before incorporation, rapeseed and milk vetch were sampled from three randomly placed 1 m^2^ quadrats in each main plot and oven-dried at 85 °C to constant weight to obtain the aboveground dry biomass. The dried samples were then ground, and their carbon and nitrogen concentrations were determined by the method of Bao [46]; the C/N ratio was calculated as the ratio of the carbon concentration to the nitrogen concentration. The carbon and nitrogen returned by each residue were calculated as the product of its aboveground dry biomass and its carbon or nitrogen concentration. The aboveground biomass (Figure 8b), C/N ratio (Figure 8c), carbon content (Figure 8d) and nitrogen content (Figure 8e) of the two residues are shown accordingly.

Theoretical grain yield was estimated for the main and ratoon crops from their yield components. At the maturity of each crop, two 1 m^2^ areas were harvested from each subplot to count the number of panicles per unit area. The panicles were threshed by hand, and the filled and unfilled spikelets were separated by immersion in water; three 30 g subsamples of filled spikelets and all of the unfilled spikelets were counted to obtain the number of spikelets per unit area and per panicle. The grain-filling percentage was taken as the ratio of the number of filled spikelets to the total number of spikelets, and the 1000-grain weight was measured with six replicates per subplot. The theoretical grain yield (t ha^−1^) of each crop was then calculated from the number of panicles per m^2^, the number of spikelets per panicle, the grain-filling percentage and the 1000-grain weight, and the annual yield was taken as the sum of the main-crop and ratoon-crop yields.

### 4.8. Statistical Analysis

Statistical analyses were carried out with SAS 9.4 (SAS Institute, Cary, NC, USA) and R 4.2.3 (https://www.r-project.org/), and all figures were generated in R. The soil properties and functional gene abundances were subjected to four-way analysis of variance to test the effects of sampling season, soil depth, cropping system and rice variety and their interactions, whereas theoretical grain yield was subjected to three-way analysis of variance involving the same factors except soil depth. Treatment means were compared using the LSD test at *p* < 0.05.

The relationships among the soil properties, functional gene abundances and theoretical grain yield were examined separately for each soil depth by Spearman’s rank correlation using the Hmisc package (version 5.1.2), and the *p* values were adjusted for multiple comparisons by the Benjamini–Hochberg procedure. Redundancy analysis was performed for each depth using the vegan package (version 2.6.4) to relate the standardised functional gene abundances to the standardised soil variables, with detrended correspondence analysis first applied to confirm that a linear method was appropriate. The significance of the full model, and of a partial model conditioned on sampling season, was assessed by permutation tests with 999 permutations, and collinearity among the explanatory variables was checked with variance inflation factors, on the basis of which STP was excluded owing to its collinearity with BD. The significant explanatory variables were identified by forward selection, and theoretical grain yield was fitted onto the ordination as a passive variable. All nine functional gene abundances were log_10_-transformed prior to the Spearman and redundancy analyses. The pathways linking cropping system, rice variety, soil physicochemical properties, functional gene abundances and the soil carbon and nitrogen pools to theoretical grain yield were analysed by partial least squares path modelling using the plspm package (version 0.4.9), with all latent variables specified in a reflective (mode A) measurement scheme. Indicators with an outer loading below 0.4 were removed from the measurement model. The structural model was evaluated from the standardised path coefficients and the *R*^2^ of the endogenous latent variables, the significance of the path coefficients was assessed with 1000 bootstrap replicates, and the overall fit of the model was evaluated with the goodness-of-fit statistic.

## 5. Conclusions

In a short-term field trial in a subtropical ratoon rice system, replacing the winter fallow with rapeseed or Chinese milk vetch raised theoretical grain yield, mainly in the ratoon season and over the full year, with little change in the main season, and reshaped the labile soil carbon and nitrogen pools and the abundances of functional genes involved in carbon and nitrogen cycling without altering the bulk physicochemical properties of the soil as a general effect. The two residues differed most in their nitrogen supply, the milk vetch with its low C/N ratio raising NH_4_^+^-N throughout the profile in the ratoon season, where it exceeded both fallow and rapeseed. These shifts were mirrored in the microbial genes, among which AOA *amoA*, *nirK* and *nirS* responded most clearly by season and depth while *narG* remained largely inert, so that the winter treatments were associated with particular steps of nitrification and denitrification rather than with the pathways as a whole. Correlation, ordination and path analyses consistently identified NH_4_^+^-N, DOC and NO_3_^−^-N as the pools most closely linked to gene abundance, and indicated that the winter treatments influenced yield indirectly through these pools rather than directly. Because the responses were consistent across the two cultivars, winter green manuring in this system need not be tailored to a specific variety, and its use in place of winter fallow merits further study, although its short duration and single location mean that the persistence of these effects warrants verification in trials spanning multiple years and sites.

## Data Availability

The raw data supporting the conclusions of this article will be made available by the authors on request.

## Supplementary Materials

The following supporting information can be downloaded at: https://www.mdpi.com/article/10.3390/plants15172578/s1, Table S1: Three-way analysis of variance (ANOVA) results on the effects of season, green manure, rice variety and their interactions on theoretical grain yield; Table S2: Four-way analysis of variance (ANOVA) results on the effects of season, soil depth, green manure, rice variety and their interactions on soil pH (pH), soil water content (SWC), soil bulk density (BD) and soil total porosity (STP); Table S3: Four-way analysis of variance (ANOVA) results on the effects of season, soil depth, green manure, rice variety and their interactions on total soil organic carbon (SOC), soil dissolved organic carbon (DOC), soil total nitrogen (STN), soil ammonium nitrogen (NH_4_^+^–N), soil nitrate nitrogen (NO_3_^−^–N) and carbon to nitrogen ratio (C/N); Table S4: Four-way analysis of variance (ANOVA) results on the effects of season, soil depth, green manure, rice variety and their interactions on the abundances of the particulate methane monooxygenase gene (*pmoA*), the methyl-coenzyme M reductase gene (*mcrA*) and the ammonia monooxygenase genes of ammonia-oxidizing archaea (AOA *amoA*) and ammonia-oxidizing bacteria (AOB *amoA*); Table S5: Four-way analysis of variance (ANOVA) results on the effects of season, soil depth, green manure, rice variety and their interactions on the abundances of the nitric oxide reductase gene (*cnorB*), the nitrate reductase gene (*narG*), the nitrite reductase genes (*nirK* and *nirS*) and the nitrous oxide reductase gene (*nosZ*); Table S6: Main soil properties at the three soil depths (0–5, 5–10 and 10–20 cm) at the beginning of the experiment; Table S7: Primers and thermal cycling conditions used for quantitative PCR of the nine functional genes.

## Abbreviations

The following abbreviations are used in this manuscript:

FA	winter fallow–ratoon rice
RA	rapeseed–ratoon rice
MV	Chinese milk vetch–ratoon rice
YLY911	Yliangyou 911
LY6326	Liangyou 6326
SE	standard error
LSD	least significant difference
SWC	soil water content
BD	soil bulk density
STP	soil total porosity
SOC	total soil organic carbon
DOC	soil dissolved organic carbon
STN	soil total nitrogen
NH_4_^+^–N	soil ammonium nitrogen
NO_3_^−^–N	soil nitrate nitrogen
C/N	carbon to nitrogen ratio
*pmoA*	particulate methane monooxygenase gene
*mcrA*	methyl-coenzyme M reductase gene
AOA *amoA*	ammonia monooxygenase genes of ammonia-oxidizing archaea
AOB *amoA*	ammonia monooxygenase genes of ammonia-oxidizing bacteria
*cnorB*	nitric oxide reductase gene
*narG*	nitrate reductase gene
*nirK* and *nirS*	nitrite reductase genes
*nosZ*	nitrous oxide reductase gene
DNA	deoxyribonucleic acid
qPCR	quantitative polymerase chain reaction
