# Supplementary material for: Responses of Soil Carbon and Nitrogen Cycling Gene Abundances to Winter Green Manure Incorporation in a Ratoon Rice System
Source: Plants (Basel). 2026 Aug 24;15(17):2578. doi: 10.3390/plants15172578 (PMC13567224; doi:10.3390/plants15172578)
Supplement: Supplementary file 1 [file plants-15-02578-s001.zip › plants-4501583-supplementary.pdf]

Supplementary Materials

Responses of Soil Carbon and Nitrogen Cycling Gene Abundances to Winter Green Manure Incorporation in a Ratoon Rice System

Supplementary Table S1. Three-way analysis of variance (ANOVA) results on the effects of season, green manure, rice variety and their interactions on theoretical grain yield.

Sources of variation	Theoretical grain yield
**Season (S)**	7.07*
**Green manure (G)**	16.07**
**Rice variety (V)**	4.79*
**S×G**	0.19ns
**S×V**	0.14ns
**G×V**	0.15ns
**S×G×V**	1.13ns

Note: Values are *F* values. ** $p < 0.01$; * $0.01 \leq p < 0.05$; ns, not significant.

Supplementary Table S2. Four-way analysis of variance (ANOVA) results on the effects of season, soil depth, green manure, rice variety and their interactions on soil pH (pH), soil water content (SWC), soil bulk density (BD) and soil total porosity (STP).

Sources of variation	pH	SWC	BD	STP
**Season (S)**	37.73**	75.59**	47.17**	47.26**
**Soil depth (D)**	0.84ns	86.73**	167.14**	165.25**
**Green manure (G)**	1.85ns	0.07ns	0.43ns	0.45ns
**Rice variety (V)**	0.00ns	1.25ns	0.76ns	0.78ns
**S×D**	0.10ns	4.45*	7.48**	7.31**
**S×G**	0.92ns	0.15ns	0.25ns	0.27ns
**S×V**	0.21ns	1.07ns	0.66ns	0.75ns
**D×G**	0.16ns	0.94ns	0.83ns	0.85ns
**D×V**	0.02ns	0.29ns	0.21ns	0.20ns
**G×V**	0.05ns	0.47ns	0.53ns	0.56ns
**S×D×G**	0.19ns	0.85ns	0.76ns	0.72ns
**S×D×V**	0.02ns	0.85ns	1.18ns	1.12ns
**S×G×V**	0.06ns	0.82ns	0.15ns	0.14ns
**D×G×V**	0.05ns	0.64ns	0.36ns	0.32ns
**S×D×G×V**	0.05ns	0.49ns	0.97ns	0.94ns

Note: Values are F values. Data from the pre-incorporation stage were not included because rice varieties had not yet been established at that time. ** $p < 0.01$; * $0.01 \le p < 0.05$; ns, not significant.

Supplementary Table S3. Four-way analysis of variance (ANOVA) results on the effects of season, soil depth, green manure, rice variety and their interactions on total soil organic carbon (SOC), soil dissolved organic carbon (DOC), soil total nitrogen (STN), soil ammonium nitrogen (NH_4^+–N), soil nitrate nitrogen (NO_3^-–N) and carbon to nitrogen ratio (C/N).

Sources of variation	SOC	DOC	STN	NH_4^+–N	NO_3^-–N	C/N
**Season (S)**	22.31**	15.42**	5.01*	57.32**	764.45**	15.58**
**Soil depth (D)**	99.88**	8.19**	13.67**	144.42**	154.38**	1.28ns
**Green manure (G)**	6.26**	8.10**	5.72**	191.02**	7.42**	2.17ns
**Rice variety (V)**	1.05ns	3.30ns	1.56ns	1.28ns	0.33ns	0.96ns
**S×D**	1.57ns	1.16ns	0.09ns	1.77ns	137.74**	0.37ns
**S×G**	0.43ns	0.14ns	0.42ns	109.97**	2.70ns	0.13ns
**S×V**	0.00ns	0.12ns	0.01ns	0.49ns	0.02ns	0.00ns
**D×G**	0.58ns	0.40ns	0.06ns	11.67**	0.43ns	0.06ns
**D×V**	0.07ns	0.78ns	0.01ns	1.01ns	0.01ns	0.03ns
**G×V**	0.04ns	0.58ns	0.03ns	0.08ns	0.08ns	0.04ns
**S×D×G**	0.36ns	0.40ns	0.09ns	3.46*	0.67ns	0.16ns
**S×D×V**	0.19ns	0.03ns	0.15ns	0.00ns	0.02ns	0.00ns
**S×G×V**	0.30ns	0.00ns	0.11ns	0.07ns	0.01ns	0.16ns
**D×G×V**	0.14ns	0.30ns	0.09ns	0.10ns	0.02ns	0.05ns
**S×D×G×V**	0.45ns	0.04ns	0.05ns	0.42ns	0.02ns	0.14ns

Note: Values are *F* values. Data from the pre-incorporation stage were not included because rice varieties had not yet been established at that time. ** $p < 0.01$; * $0.01 \le p < 0.05$; ns, not significant.

Supplementary Table S4. Four-way analysis of variance (ANOVA) results on the effects of season, soil depth, green manure, rice variety and their interactions on the abundances of the particulate methane monooxygenase gene (*pmoA*), the methyl-coenzyme M reductase gene (*mcrA*) and the ammonia monooxygenase genes of ammonia-oxidizing archaea (AOA *amoA*) and ammonia-oxidizing bacteria (AOB *amoA*).

**Sources of variation**	***pmoA***	***mcrA***	**AOA *amoA***	**AOB *amoA***
**Season (S)**	142.77**	76.81**	90.03**	33.12**
**Soil depth (D)**	26.53**	35.93**	79.43**	35.59**
**Green manure (G)**	31.23**	22.95**	20.72**	3.72*
**Rice variety (V)**	1.22ns	9.51**	18.63**	0.21ns
**S×D**	4.05*	0.19ns	5.26**	2.90ns
**S×G**	7.94**	8.97**	0.82ns	0.07ns
**S×V**	3.06ns	4.62*	0.12ns	0.03ns
**D×G**	3.46*	7.31**	4.88**	0.40ns
**D×V**	2.06ns	4.04*	1.01ns	0.99ns
**G×V**	1.55ns	1.26ns	2.06ns	0.58ns
**S×D×G**	2.23ns	0.21ns	2.35ns	0.22ns
**S×D×V**	0.52ns	0.28ns	12.76**	0.54ns
**S×G×V**	4.93**	1.86ns	11.57**	0.87ns
**D×G×V**	0.56ns	2.84*	1.30ns	0.62ns
**S×D×G×V**	1.74ns	0.41ns	3.56*	0.32ns

Note: Values are F values. Data from the pre-incorporation stage were not included because rice varieties had not yet been established at that time. ** $p < 0.01$; * $0.01 \le p < 0.05$; ns, not significant.

Supplementary Table S5. Four-way analysis of variance (ANOVA) results on the effects of season, soil depth, green manure, rice variety and their interactions on the abundances of the nitric oxide reductase gene (*cnorB*), the nitrate reductase gene (*narG*), the nitrite reductase genes (*nirK* and *nirS*) and the nitrous oxide reductase gene (*nosZ*).

Sources of variation	*cnorB*	*narG*	*nirK*	*nirS*	*nosZ*
**Season (S)**	6.37*	23.37**	66.97**	96.46**	6.78*
**Soil depth (D)**	11.56**	1.94ns	24.93**	3.57*	12.05**
**Green manure (G)**	3.37*	1.56ns	97.02**	3.38*	14.67**
**Rice variety (V)**	2.43ns	2.64ns	6.29*	2.67ns	6.73*
**S×D**	0.36ns	0.89ns	5.22**	0.72ns	40.10**
**S×G**	0.72ns	1.31ns	7.50**	0.93ns	13.45**
**S×V**	0.07ns	1.31ns	19.77**	0.21ns	0.57ns
**D×G**	0.12ns	0.10ns	9.20**	0.03ns	2.38ns
**D×V**	0.03ns	0.04ns	0.69ns	0.23ns	1.05ns
**G×V**	0.11ns	0.51ns	0.21ns	0.03ns	0.05ns
**S×D×G**	0.38ns	0.19ns	8.23**	0.12ns	5.46**
**S×D×V**	0.16ns	0.01ns	7.36**	0.04ns	0.11ns
**S×G×V**	0.05ns	0.86ns	4.84*	0.17ns	0.10ns
**D×G×V**	0.25ns	0.04ns	4.50**	0.16ns	1.49ns
**S×D×G×V**	0.28ns	0.13ns	0.37ns	0.16ns	2.18ns

Note: Values are *F* values. Data from the pre-incorporation stage were not included because rice varieties had not yet been established at that time. ** $p < 0.01$; * $0.01 \le p < 0.05$; ns, not significant.

Supplementary Table S6. Main soil properties at the three soil depths (0–5, 5–10 and 10–20 cm) at the beginning of the experiment.

The main soil properties	Soil depths		
	0–5 cm	5–10 cm	10–20 cm
**Soil pH**	4.62 ± 0.21	4.90 ± 0.16	5.08 ± 0.04
**Soil bulk density (g cm^{-3})**	1.22 ± 0.03	1.21 ± 0.00	1.39 ± 0.02
**Soil total porosity (%)**	54.56 ± 1.49	54.44 ± 0.15	47.62 ± 0.74
**Total soil organic carbon (g kg^{-1})**	14.53 ± 0.04	14.37 ± 0.10	13.99 ± 0.16
**Soil total nitrogen (g kg^{-1})**	1.06 ± 0.08	1.05 ± 0.07	1.02 ± 0.11
**Soil total phosphorus (g kg^{-1})**	1.05 ± 0.02	1.04 ± 0.01	0.98 ± 0.03
**Soil total potassium (g kg^{-1})**	18.17 ± 1.51	17.33 ± 0.27	17.31 ± 0.12
**Soil ammonium nitrogen (mg kg^{-1})**	20.80 ± 1.22	15.65 ± 0.90	11.76 ± 0.84
**Soil nitrate nitrogen (mg kg^{-1})**	0.85 ± 0.16	0.62 ± 0.17	0.51 ± 0.03
**Soil available phosphorus (mg kg^{-1})**	134.12 ± 6.03	130.99 ± 12.55	130.56 ± 10.48
**Soil available potassium (mg kg^{-1})**	130.61 ± 8.16	80.00 ± 3.33	74.44 ± 7.29

Note: Values are means ± standard error of three replicates.

Supplementary Table S7. Primers and thermal cycling conditions used for quantitative PCR of the nine functional genes.

Target gene	Primer	Sequences (5′–3′)	qPCR programme	Reference
pmoA	325F<br>643R	TGGGGYTGGACCTAYTTCC;<br>CCGGCRCRACGTCCTTACC	95 °C, 5 min; 50 cycles of (95 °C, 30 s; 55 °C, 35 s; 72 °C, 30 s with plate read); melt curve 65–95 °C, 0.5 °C increment	[1]
mcrA	qmcrA-F<br>qmcrA-R	TTCGGTGGATCDCARAGRGC;<br>GBARGTCGWAWCCGTAGAATCC	95 °C, 5 min; 50 cycles of (95 °C, 30 s; 60 °C, 35 s; 72 °C, 30 s with plate read); melt curve 65–95 °C, 0.5 °C increment	[2]
**AOA *amoA***	Arch-amoAF<br>Arch-amoAR	STAATGGTCTGGCTTAGACG;<br>GCGGCCATCCATCTGTATGT	95 °C, 5 min; 70 cycles of (95 °C, 30 s; 58 °C, 50 s; 72 °C, 30 s with plate read); melt curve 65–95 °C, 0.5 °C increment	[3]
**AOB *amoA***	amoA-1F<br>amoA-2R	GGGGTTTCTACTGGTGGT;<br>CCCCTCKGSAAAGCCTTCTTC	95 °C, 5 min; 50 cycles of (95 °C, 30 s; 55 °C, 35 s; 72 °C, 30 s with plate read); melt curve 65–95 °C, 0.5 °C increment	[4]
cnorB	cnorBBF<br>cnorBBR	AIGTGGTCGAGAAGTGGCTCTA;<br>TCTGIACGGTGAAGATCACC	95 °C, 5 min; 50 cycles of (95 °C, 30 s; 55 °C, 35 s; 72 °C, 30 s with plate read); melt curve 65–95 °C, 0.5 °C increment	[5]
narG	narG2F<br>narG2R	TAYGTSGGGCAGGARAAACTG;<br>CGTAGAAGAAGCTGGTGCTGTT	95 °C, 5 min; 50 cycles of (95 °C, 30 s; 55 °C, 35 s; 72 °C, 30 s with plate read); melt curve 65–95 °C, 0.5 °C increment	[4]
nirK	nirK1F<br>nirK5R	GGMATGGTKCCSTGGCA;<br>GCCTCGATCAGRTTRTGG	95 °C, 5 min; 50 cycles of (95 °C, 30 s; 61 °C, 40 s; 72 °C, 30 s with plate read); melt curve 65–95 °C, 0.5 °C increment	[6]
nirS	cd3aF<br>R3cd	GTNAAYGTNAARGARACNGG;<br>GASTTCGGRTGSGTCTTGA	95 °C, 5 min; 60 cycles of (95 °C, 30 s; 58 °C, 50 s; 72 °C, 30 s with plate read); melt curve 65–95 °C, 0.5 °C increment	[4]
nosZ	nosZ2F<br>nosZ2R	CGCRACGGCAASAAGGTSMSSGT;<br>CAKRTGCAKSGCRTGGCAGAA	95 °C, 5 min; 50 cycles of (95 °C, 30 s; 64 °C, 35 s; 72 °C, 30 s with plate read); melt curve 65–95 °C, 0.5 °C increment	[4]

References

1. Yu, X.; Yang, X.; Wu, Y.; Peng, Y.; Yang, T.; Xiao, F.; Zhong, Q.; Xu, K.; Shu, L.; He, Q. Sonneratia apetala introduction alters methane cycling microbial communities and increases methane emissions in mangrove ecosystems. *Soil Biol. Biochem.* **2020**, *144*, 107775.
2. Denman, S.E.; Tomkins, N.W.; McSweeney, C.S. Quantitation and diversity analysis of ruminal methanogenic populations in response to the antimethanogenic compound bromochloromethane. *FEMS Microbiol. Ecol.* **2007**, *62*, 313-322.
3. Francis, C.A.; Roberts, K.J.; Beman, J.M.; Santoro, A.E.; Oakley, B.B. Ubiquity and diversity of ammonia-oxidizing archaea in water columns and sediments of the ocean. *Proc. Natl. Acad. Sci. U.S.A.* **2005**, *102*, 14683-14688.
4. Fang, W.; Yan, D.; Huang, B.; Ren, Z.; Wang, X.; Liu, X.; Li, Y.; Ouyang, C.; Migheli, Q.; Cao, A. Biochemical pathways used by microorganisms to produce nitrous oxide emissions from soils fumigated with dimethyl disulfide or allyl isothiocyanate. *Soil Biol. Biochem.* **2019**, *132*, 1-13.
5. Dandie, C.; Burton, D.; Zebarth, B.; Trevors, J.; Goyer, C. Analysis of denitrification genes and comparison of nosZ, cnorB and 16S rDNA from culturable denitrifying bacteria in potato cropping systems. *Syst. Appl. Microbiol.* **2007**, *30*, 128-138.
6. Braker, G.; Schwarz, J.; Conrad, R. Influence of temperature on the composition and activity of denitrifying soil communities. *FEMS Microbiol. Ecol.* **2010**, *73*, 134-148.
